# Developmentally Guided Differentiation of Ventral Midbrain Dopaminergic Cells Validated in Proteopathy‐Based Parkinson's Disease Models

**DOI:** 10.1002/advs.78007

**Published:** 2026-09-29

**Authors:** Heechang Moon, Heejeong Kim, Elliot H. Lee, Hanseul Kim, Ji Hun Kim, Huisu Jeong, Jiwon Cheon, Ga Ram Jeong, Byoung Dae Lee, Jae‐Won Cho, Soo‐Jin Oh, Yunjong Lee, Yohan Oh

**Affiliations:** ^1^ Department of Biomedical Science Graduate School of Biomedical Science and Engineering Hanyang University Seoul Republic of Korea; ^2^ Department of Pharmacology Samsung Biomedical Research Institute Sungkyunkwan University School of Medicine Suwon Republic of Korea; ^3^ Bio Healthcare Central Research Center Hyper Corporation Inc. Seoul Republic of Korea; ^4^ Brain Science Institute Korea Institute of Science and Technology Seoul Republic of Korea; ^5^ Department of Biotechnology College of Life Science and Biotechnology Yonsei University Seoul Republic of Korea; ^6^ Hanyang Institute of Bioscience and Biotechnology Hanyang University Seoul Republic of Korea; ^7^ Department of Neuroscience Kyung Hee University Seoul Republic of Korea; ^8^ Department of Physiology Kyung Hee University Seoul Republic of Korea; ^9^ Department of Life Science College of Natural Sciences Hanyang University Seoul Republic of Korea; ^10^ Department of Biochemistry and Molecular Biology College of Medicine Hanyang University Seoul Republic of Korea; ^11^ Hanyang Institute of Advanced BioConvergence Hanyang University Seoul Republic of Korea

**Keywords:** cell therapy, differentiation protocol, human pluripotent stem cell, Parkinson's disease, ventral midbrain dopaminergic neuron, ventral midbrain dopaminergic progenitor

## Abstract

Parkinson's disease (PD) involves progressive degeneration of nigrostriatal ventral midbrain dopaminergic (vmDA) neurons and Lewy body proteopathy, necessitating scalable, lineage‐faithful donor cells. However, variability in human pluripotent stem cell (hPSC) differentiation and incomplete ventral midbrain identity limit translational use. Here, we establish a developmentally guided differentiation protocol that reproducibly specifies EN1‐positive vmDA progenitors through temporally optimized SHH, WNT/β‐catenin, and FGF8 signaling. The resulting progenitors mature into vmDA neurons exhibiting stimulus‐dependent dopamine release and maturation‐associated pacemaking activity. Progenitors can be cryopreserved at a defined stage while retaining viability and neurogenic capacity. Functional validation was performed using complementary proteopathy‐based PD models. In vitro, optogenetic induction of α‐synuclein aggregation (*OASIS*) triggered robust aggregate formation and selective degeneration of TH‐positive vmDA neurons in both two‐dimensional cultures and three‐dimensional neurospheroids. In vivo, we established a conditional β23 proteopathy‐based PD mouse model exhibiting progressive nigrostriatal degeneration and motor impairment. Transplantation of hESC‐derived vmDA progenitors into advanced‐stage PD mice led to graft survival, differentiation into vmDA neurons, and significant motor improvement. Together, these findings define a robust vmDA differentiation framework validated across proteopathy‐based disease modeling and transplantation contexts, supporting its application in PD modeling and cell therapy.

## Graphical abstract

A developmentally guided protocol reproducibly specifies EN1‐positive ventral midbrain dopaminergic (vmDA) progenitors from human pluripotent stem cells and yields functional vmDA neurons. Optogenetic induction of α‐synuclein aggregation elicits selective degeneration of TH‐positive neurons in 2D cultures and 3D neurospheroids, while transplanted progenitors survive, differentiate into vmDA neurons, and improve motor function in a proteopathy‐based Parkinson's disease mouse model.

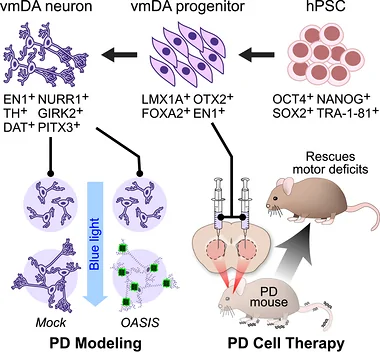

## 1 Introduction

Parkinson's disease (PD) is the second most common neurodegenerative disorder and is pathologically defined by the progressive degeneration of nigrostriatal ventral midbrain dopaminergic (vmDA) neurons in the substantia nigra pars compacta (SNpc), accompanied by Lewy body proteopathy [1]. The resulting disruption of nigrostriatal dopamine signaling leads to core motor symptoms, including bradykinesia, rigidity, and resting tremor. Although current therapeutic strategies—such as dopaminergic pharmacotherapy, deep brain stimulation, and gene‐based interventions—can alleviate symptoms, none halt or reverse the underlying neurodegenerative process [1]. Cell replacement therapy has therefore emerged as a promising disease‐modifying approach for PD. Early clinical studies using fetal ventral mesencephalic tissue demonstrated that transplanted dopaminergic neurons can survive and function in the PD brain [2]. However, ethical concerns regarding fetal tissue procurement, limited scalability, variability in graft composition, and insufficient control over cell identity have restricted broader clinical application [3].

Human pluripotent stem cells (hPSCs), including human embryonic stem cells (hESCs) and human induced pluripotent stem cells (hiPSCs), provide a renewable source of dopaminergic lineage cells for transplantation‐based strategies [4]. In particular, vmDA progenitors derived from hPSCs have been explored as donor cells for PD, based on their ventral midbrain regional identity and their capacity to mature into dopaminergic neurons after engraftment [1]. Over the past decade, numerous differentiation protocols have been reported to generate vmDA lineage cells by modulating developmental signaling pathways, including sonic hedgehog (SHH), fibroblast growth factor 8 (FGF8), and WNT/β‐catenin signaling [1, 5, 6, 7, 8, 9, 10]. However, many reported protocols yield vmDA populations with low differentiation efficiency or heterogeneous expression of key vmDA markers such as EN1, NURR1, and TH [1]. This variability complicates the generation of authentic vmDA progenitors and neurons with consistent identity and quality.

In this study, we developed a developmentally guided differentiation protocol that enables robust generation of vmDA lineage cells from hPSCs. The protocol improves both efficiency and reproducibility of vmDA specification by sustaining SHH signaling and temporally gating FGF8 activation, yielding vmDA progenitors and vmDA neurons that express established vmDA markers and exhibit functional properties associated with vmDA neurons. To validate the authenticity of vmDA neurons produced by the protocol, we examined whether these neurons could model a defining pathological feature of PD in vitro. Specifically, we applied an optogenetics‐assisted α‐synuclein (α‐syn) aggregation induction system (*OASIS*), in which exposure to blue light triggers aggregation of α‐synuclein [11]. Using this system, α‐syn aggregation was induced in hPSC‐derived vmDA neurons cultured in both two‐dimensional monolayers and three‐dimensional neurospheroid formats, leading to degeneration of TH‐positive vmDA neurons. This approach allowed us to assess whether vmDA neurons generated by the protocol exhibit proteopathy‐associated vulnerability consistent with vmDA neuron loss in PD. In parallel, we evaluated the authenticity of hPSC‐derived vmDA progenitors in a transplantation context. The vmDA progenitors intended for cell therapy must survive after engraftment, differentiate into vmDA neurons in vivo, and contribute to functional improvement. To assess these properties under proteopathy‐associated disease conditions, we developed such a PD mouse model and transplanted hESC‐derived vmDA progenitors into the striatum. Post‐transplantation outcomes were assessed using histological analysis of graft‐derived vmDA neurons and motor behavior assays.

Together, this study reports a new hPSC‐based vmDA differentiation protocol and demonstrates that vmDA progenitors and neurons generated by this protocol represent authentic vmDA cell states. By validating vmDA neurons through in vitro modeling of PD‐associated α‐syn aggregation and TH‐positive neuron degeneration, and independently validating vmDA progenitors through transplantation into a PD mouse model, we establish the fidelity of the differentiation protocol for both PD disease modeling and cell therapy‐oriented studies.

## 2 Results

### 2.1 Developmentally Guided Optimization of vmDA Progenitor Specification

Efficient induction of vmDA lineage cells from hPSCs requires faithful recapitulation of early neural and regional patterning cues. Accordingly, all differentiation protocols used in this study were initiated with dual‐SMAD inhibition to establish a homogeneous neuroectodermal baseline [5], followed by systematic optimization of ventral midbrain patterning signals. In the developing ventral midbrain, vmDA progenitors arise from the floor plate and are defined by the co‐expression of LMX1A and FOXA2 [12, 13]; therefore, LMX1A/FOXA2 co‐expression was used as the primary indicator of vmDA progenitor commitment. Starting from a previously reported vmDA differentiation framework [14], we first optimized SHH and WNT/β‐catenin signaling strength. Increasing SHH concentration enhanced the proportion of LMX1A/FOXA2 double‐positive progenitors, with diminishing returns above 100 ng/mL and no corresponding increase at the transcript level; thus, 100 ng/mL SHH was selected based on effect size and cost‐effectiveness (Figure S1A–D). Titration of the WNT activator CHIR99021 (CHIR) identified 3 µm as the concentration yielding the highest proportion of LMX1A‐ and FOXA2‐expressing progenitors (Figure S1E–H). Because FGF8 signaling plays a central role in vmDA fate specification [8], we next examined its temporal requirement. Late‐stage FGF8 exposure significantly increased LMX1A/FOXA2 progenitor yield and subsequently enhanced TH‐expressing neuronal output, indicating that temporally restricted FGF8 signaling contributes to efficient vmDA lineage progression (Figure S1I–N). Despite these optimizations, LMX1A induction remained relatively limited within the FOXA2‐expressing progenitor population. Given the established role of WNT–LMX1A regulatory circuitry in stabilizing vmDA identity [13], we therefore optimized the timing of pathway activation (Figure S2A). Advancing WNT activation from DIV 3 to DIV 0 selectively enhanced LMX1A expression, whereas advancing SHH/PMP treatment from DIV 1 to DIV 0 reduced LMX1A expression despite modest FOXA2 induction (Figure S2B–D). Although simultaneous early activation of both pathways produced comparable marker profiles, it resulted in greater batch‐to‐batch variability (Figure S2E); thus, early WNT activation alone was selected to maximize robustness and reproducibility. Consistent with prior reports [15], a transient early CHIR boost further enhanced LMX1A expression, and a single optimized boost outperformed sequential boosting strategies (Figure S2F–I). For translational applicability, the optimized patterning strategy was adapted to feeder‐free, KSR‐free conditions. Removal of KSR necessitated re‐optimization of CHIR concentration [16], and at 0.8 µm CHIR, *OTX2* expression was modestly reduced relative to the 0 µm condition representing anterior (forebrain/midbrain) neural identity, while the hindbrain marker *GBX2* was not induced (Figure S2J,K). This expression pattern is consistent with initiation of midbrain regionalization, and 0.8 µm CHIR was selected for subsequent differentiation. Under KSR‐free conditions, Neurobasal medium supplemented either with B27 or with N2 and B27 yielded comparable proportions of LMX1A‐ and FOXA2‐expressing progenitors with high reproducibility. Because the inclusion of N2 did not confer a measurable advantage in progenitor yield or consistency, Neurobasal medium supplemented with B27 was selected as a more streamlined formulation for subsequent experiments (Figure S2L–O). However, despite improved progenitor specification, terminal neuronal differentiation under baseline conditions yielded limited numbers of TH‐expressing neurons, and extension of FGF8 exposure produced only modest improvement (Figure S3A,B).

To further enhance vmDA neuronal output, we next optimized the maturation context of differentiated progenitors. Three‐dimensional (3D) neurosphere‐based maturation markedly increased the generation of TH‐ and TUJ1‐expressing neurons compared with two‐dimensional monolayer culture (Figure 1 and Figure S3C,D). However, additional modulation of FGF8 signaling, including biphasic or late‐stage treatment, failed to induce robust expression of the essential midbrain identity marker EN1 and was accompanied by reduced TH expression (Figure S3E–J). These findings indicate that FGF8 modulation alone is insufficient to establish complete vmDA identity, prompting us to investigate alternative patterning strategies to enhance EN1 expression in vmDA progenitors.

**FIGURE 1:**
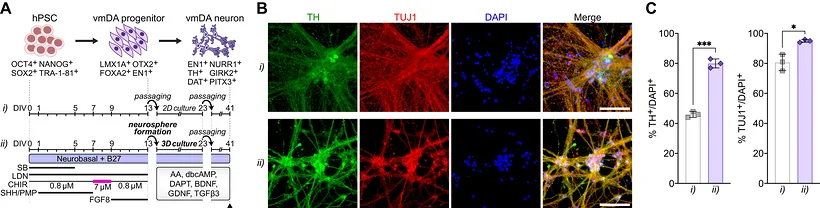
Neurosphere‐based maturation enhanced dopaminergic differentiation of vmDA progenitors. (A) Schematic overview of the differentiation workflow comparing two‐dimensional (2D) monolayer culture and three‐dimensional (3D) neurosphere‐based maturation. (B) Representative immunofluorescence images of differentiated neurons at DIV 41 stained for TH (green) and TUJ1 (red); nuclei were counterstained with DAPI (blue). (C) Quantification of TH^+^ and TUJ1^+^ cells expressed as a percentage of total DAPI^+^ cells (*n* = 3 biological replicates). An arrowhead indicates the time point for downstream experiments. Scale bars, 100 µm. Data are presented as mean ± SD. Statistical significance was assessed by two‐tailed unpaired Student's *t*‐test. ^*^ *p* < 0.05, ^***^ *p* < 0.001.

### 2.2 Sustained SHH Signaling Promotes EN1 Induction During vmDA Progenitor Specification

Based on a prior study indicating that insufficient SHH signaling leads to impaired midbrain dopaminergic neurogenesis [17], we examined whether sustained activation of SHH signaling during the patterning phase could enhance EN1 induction under the reference patterning conditions (Figure 2A). To this end, we extended the duration of treatment with PMP, a chemical agonist of the SHH pathway, either alone or in combination with SHH ligand. RT‐qPCR analysis revealed that extension of PMP treatment alone increased the expression of *EN1* and *LMX1A* relative to the reference protocol, while *FOXA2* expression remained unchanged. In contrast, concomitant extension of both SHH and PMP treatment resulted in a further increase in *EN1* and *LMX1A* expression and additionally elevated *FOXA2* expression, yielding the highest overall expression levels among the tested conditions (Figure 2B). At the cellular level, immunocytochemical analysis revealed that extension of PMP treatment alone or in combination with SHH markedly increased the proportion of EN1‐expressing progenitors to approximately 50%, whereas the proportions of progenitors expressing OTX2, LMX1A, or FOXA2 remained unchanged relative to the reference protocol, consistent with their already widespread expression under the established differentiation conditions (Figure 2C–F). Given the positive effect of sustained SHH signaling on EN1 induction, we next extended the duration of the patterning phase to further enhance vmDA progenitor identity (Figure 2G). Extension of the patterning phase beyond the standard endpoint (DIV 13) to DIV 14, DIV 15, and DIV 16 led to a progressive, duration‐dependent increase in the proportion of EN1‐expressing progenitors, with the highest EN1‐positive yield observed at the longest patterning duration (Figure 2H–K).

**FIGURE 2:**
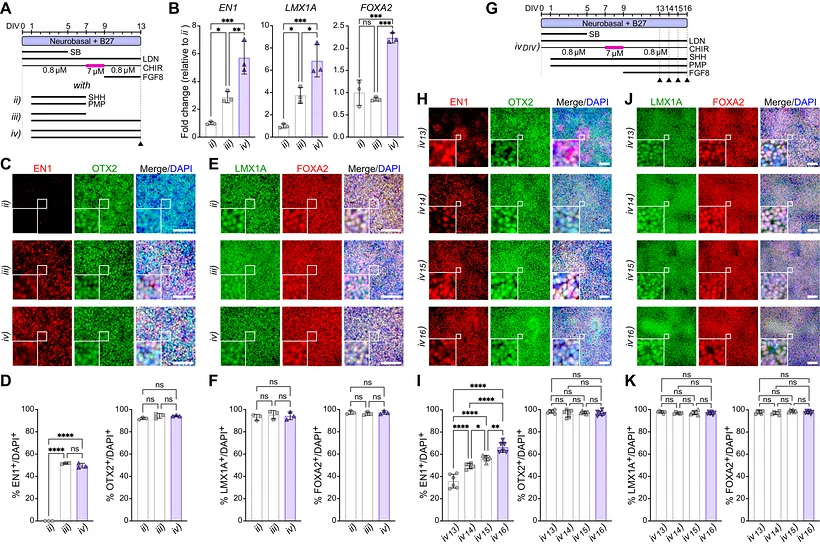
Sustained SHH signaling promoted EN1 induction during vmDA progenitor specification. (A) Schematic comparison of vmDA differentiation protocols with differential duration of SHH pathway activation. (B) RT‐qPCR analysis of *EN1*, *LMX1A*, and *FOXA2* expression at DIV 13, shown as fold change relative to protocol *ii* (*n* = 3 biological replicates). (C, E) Representative immunofluorescence images of DIV 13 progenitors stained for EN1 (red) and OTX2 (green) (C) or LMX1A (green) and FOXA2 (red) (E); nuclei were counterstained with DAPI (blue). Insets show enlarged views of the boxed regions. (D, F) Quantification of marker‐positive cells among total DAPI^+^ cells (*n* = 3 biological replicates). (G) Schematic illustrating extended patterning duration. (H, J) Representative immunofluorescence images of progenitors at DIV 13–16 stained for EN1 (red) and OTX2 (green) (H) or LMX1A (green) and FOXA2 (red) (J); nuclei were counterstained with DAPI (blue). Insets show enlarged views of the boxed regions. (I, K) Quantification of marker‐positive cells among total DAPI^+^ cells at each differentiation stage (*n* = 6 biological replicates). Arrowheads indicate the time points for downstream experiments. Scale bars: 100 µm. Data are mean ± SD. Statistical significance was determined by one‐way ANOVA with Tukey's multiple‐comparison test. ns, not significant; ^*^ *p* < 0.05, ^**^ *p* < 0.01, ^***^ *p* < 0.001, ^****^ *p* < 0.0001.

To validate the performance of the vmDA differentiation strategy incorporating the indicated modifications, we next performed a direct comparison with the original reference protocol that served as the basis for our modifications (Figure S4A). RT‐qPCR analysis demonstrated that the modified protocol significantly increased the expression of key vmDA identity markers, including *EN1*, *LMX1A*, and *FOXA2*, at DIV 5, corresponding to the early progenitor stage (Figure S4B). Enhanced expression of these markers was maintained through DIV 16, the final day of the patterning phase (Figure S4C). Notably, *TH* expression was also increased at DIV 16 under the modified protocol, whereas no difference was observed at DIV 5, indicating that improved vmDA progenitor identity preceded enhanced dopaminergic differentiation output. At a later stage corresponding to vmDA neuronal differentiation (DIV 41), expression of *EN1*, *LMX1A*, and *FOXA2* remained significantly higher under the modified protocol, and *TH* expression was also elevated relative to the reference condition (Figure S4D).

### 2.3 Temporal Optimization of CHIR Boosting Maximizes EN1‐ and LMX1A‐expressing vmDA Progenitors

Following the successful enhancement of EN1‐expressing vmDA progenitors by extending the patterning phase, we next investigated whether further optimization of WNT signaling through temporal adjustment of CHIR boosting could increase the yield of EN1‐positive progenitors (Figure 3A). Comparative analysis revealed that CHIR boosting applied specifically between DIV 11 and DIV 13 produced the highest proportion of EN1‐ and LMX1A‐expressing progenitors among all tested conditions (Figure 3B,C). After identifying the optimal timing window for CHIR boosting, we next examined whether extending the duration of CHIR exposure could further improve progenitor yield (Figure 3D). Continuous CHIR boosting maintained through DIV 16 resulted in the maximal enrichment of EN1‐ and LMX1A‐expressing progenitors, achieving approximately 90% double‐positive cells under this condition (Figure 3E,F).

**FIGURE 3:**
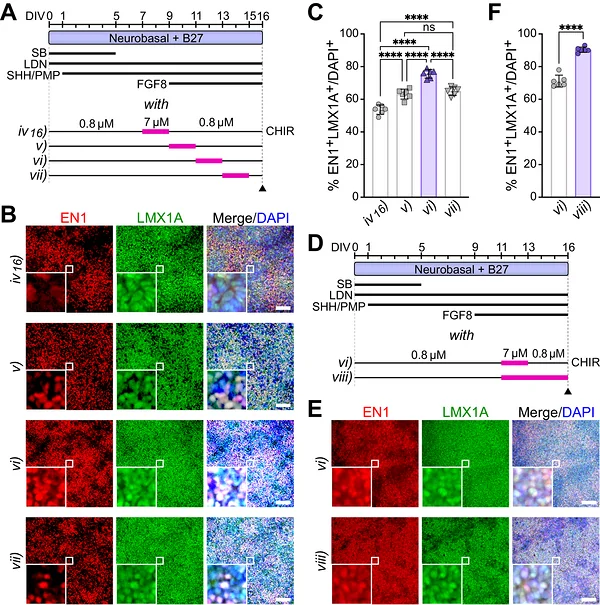
Temporal optimization of CHIR99021 boosting maximized EN1^+^/LMX1A^+^ vmDA progenitor yield. (A, D) Schematics illustrating differentiation protocols with distinct timing and duration of CHIR99021 boosting. (B, E) Representative immunofluorescence images of DIV 16 vmDA progenitors stained for EN1 (red) and LMX1A (green); nuclei were counterstained with DAPI (blue). Insets show enlarged views of the boxed regions. (C, F) Quantification of EN1/LMX1A double‐positive progenitors among total DAPI^+^ cells (*n* = 6 biological replicates). Arrowheads indicate the time points for downstream experiments. Scale bars: 100 µm. Data are mean ± SD. Statistical significance was assessed by one‐way ANOVA with Tukey's multiple‐comparison test (C) or two‐tailed unpaired Student's *t*‐test (F). ns, not significant; ^****^ *p* < 0.0001.

### 2.4 Molecular and Functional Maturation of vmDA Neurons Generated by the Optimized Protocol

Through sequential optimization of developmental signaling parameters, we established a highly efficient vmDA neuron differentiation protocol (Figure 4A). RT‐qPCR analysis revealed that early vmDA progenitor markers, including *EN1*, *LMX1A*, and *FOXA2*, were significantly upregulated at DIV 16 compared with DIV 0 (Figure 4B). In contrast, markers associated with more mature dopaminergic stages, such as *NURR1* and *TH*, showed prominent induction at DIV 26, and the elevated expression of both early‐ and late‐stage markers was maintained through DIV 34. Notably, expression of the hindbrain marker *KROX20* remained unchanged relative to DIV 0, indicating preservation of ventral midbrain identity. This temporal expression pattern is consistent with the in vivo vmDA developmental trajectory [18]. We next compared the optimized protocol with the vmDA differentiation protocol developed by Kim et al. [15], which introduced a biphasic WNT activation strategy to facilitate scalable generation of vmDA neurons for translational use (Figure S5A,B). At DIV 16, corresponding to the vmDA progenitor stage, the optimized protocol yielded significantly higher *EN1* and *LMX1A* expression than the Kim et al. protocol, whereas no significant differences in *FOXA2* or *TH* expression were detected between the protocols (Figure S5C). At DIV 40, corresponding to the neuronal differentiation stage, the optimized protocol yielded significantly higher expression of *EN1*, *LMX1A*, *FOXA2*, and *TH* than the Kim et al. protocol (Figure S5D). Together, these data indicate that the optimized protocol yielded higher *EN1* and *LMX1A* expression at the progenitor stage and broader increases in vmDA/dopaminergic marker expression at the neuronal stage relative to the Kim et al. protocol.

**FIGURE 4:**
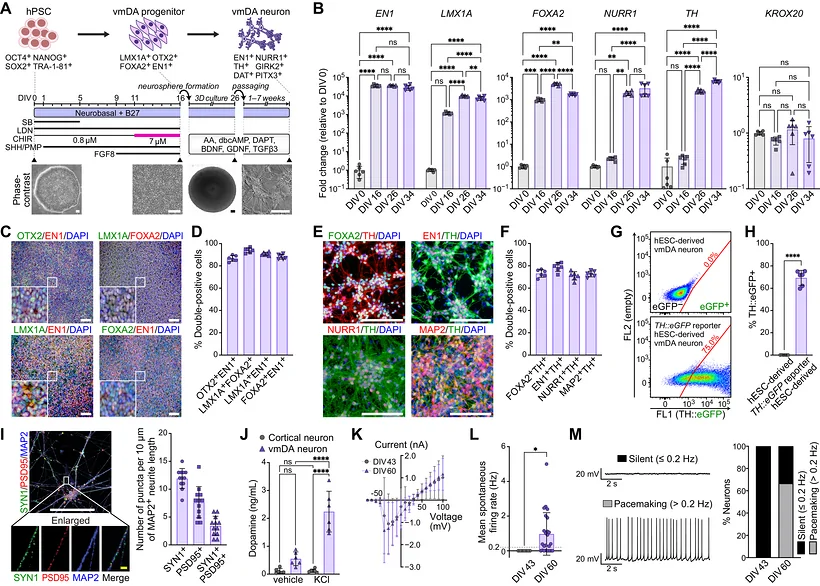
Optimized differentiation protocol generated molecularly and functionally mature human vmDA neurons. (A) Schematic of the optimized vmDA differentiation protocol and representative phase‐contrast images at indicated stages. (B) RT‐qPCR analysis of vmDA lineage markers (*EN1*, *LMX1A*, *FOXA2*, *NURR1*, *TH*) and the hindbrain marker *KROX20* at indicated differentiation stages, expressed as fold change relative to DIV 0 (*n* = 6 biological replicates). (C, D) Representative immunofluorescence images and quantification of cells double‐positive for the indicated vmDA progenitor markers among total DAPI^+^ cells at DIV 16 (*n* = 6 biological replicates). Insets in C show enlarged views of the boxed regions. (E, F) Representative images and quantification of cells double‐positive for TH and the indicated vmDA or neuronal markers among total DAPI^+^ cells at DIV 39 (*n* = 6 biological replicates). (G, H) Flow cytometric analysis and quantification of TH::eGFP^+^ cells at DIV 39 following vmDA neuronal differentiation (*n* = 6 biological replicates). (I) Synaptic maturation at DIV 70 visualized by SYN1 (green), PSD95 (red), and MAP2 (blue) immunostaining, with an enlarged view of the boxed region shown below (left). Quantification of synaptic puncta along MAP2^+^ neurites (right; *n* = 12 neurite segments from 3 biological replicates). (J) Release of dopamine from vmDA neurons (DIV 35) and cortical neurons (DIV 35) after vehicle or 50 mm KCl treatment, analyzed by ELISA (*n* = 6 biological replicates). (K–M) Whole‐cell patch‐clamp electrophysiological characterization of vmDA neurons at DIV 43 and DIV 60. (K) Current–voltage relationships (*n* = 6 neurons from 3 biological replicates). (L) Spontaneous firing rate, calculated over 1 min (*n* = 7 neurons at DIV 43 and *n* = 24 neurons at DIV 60, obtained from 3 biological replicates per stage). (M) Example traces of silent (≤ 0.2 Hz) and pacemaking (> 0.2 Hz) neurons (left) and proportions across indicated DIVs (*n* = 7 neurons at DIV 43 and *n* = 24 neurons at DIV 60, obtained from 3 biological replicates per stage) (right). Arrowheads indicate the time points for downstream experiments. Scale bars: 100 µm (white) and 2 µm (yellow). Data are mean ± SD. Statistical analyses were performed using one‐way ANOVA (B) or two‐way ANOVA (J) with Tukey's multiple‐comparison test, or two‐tailed unpaired Student's *t*‐test (H, L). ns, not significant; ^*^ *p* < 0.05, ^**^ *p* < 0.01, ^***^ *p* < 0.001, ^****^ *p* < 0.0001.

Consistent with these transcriptional analyses, immunocytochemical evaluation confirmed the generation of vmDA progenitors with high purity at DIV 16 (Figure 4C,D and Figure S5E). Upon further maturation, approximately 70–80% of cells were double‐positive for TH and established dopaminergic or neuronal markers, including FOXA2, EN1, NURR1, and MAP2, indicating efficient differentiation toward a vmDA neuronal phenotype (Figure 4E,F). These observations were also confirmed by flow cytometric quantification of TH::TH‐T2A‐eGFP (TH::eGFP)‐positive cells following differentiation of a *TH::eGFP* reporter hESC line into vmDA neurons (Figure 4G,H). At later stages (DIV 46 and DIV 61), expression of mature vmDA neuronal markers, including PITX3, DAT, and GIRK2, was detected, confirming progression toward a mature vmDA phenotype (Figure S5F,G). At DIV 61, TH‐positive and GIRK2‐positive cells accounted for approximately 74% and 68% of total DAPI‐positive cells, respectively. Reciprocal co‐expression analysis further showed that approximately 91% of TH‐positive cells expressed GIRK2 and that approximately 99% of GIRK2‐positive cells co‐expressed TH (Figure S5G). Morphological maturation was further supported by the formation of robust synaptic structures at DIV 70 (Figure 4I). In addition, ELISA analysis detected dopamine release from vmDA neurons at DIV 35 in response to depolarization with 50 mm KCl, demonstrating stimulus‐dependent dopaminergic activity (Figure 4J).

To directly assess electrophysiological maturation, we performed whole‐cell patch‐clamp recordings at DIV 43 and DIV 60. Resting membrane potential and input resistance did not differ significantly between stages, whereas cell capacitance was significantly higher at DIV 60 (Figure S5H–J). Although the I–V relationship showed no marked difference (Figure 4K), evoked firing responses revealed qualitative maturation of action potential properties, with DIV 60 neurons exhibiting more mature action potential waveforms and higher firing frequencies across a range of current injections compared with DIV 43 neurons (Figure S5K,L). Spontaneous firing activity assessed under current‐clamp gap‐free conditions demonstrated a clear maturation‐dependent transition in firing behavior, with all recorded neurons remaining silent at DIV 43, whereas approximately 67% of neurons exhibited pacemaking activity at DIV 60 (Figure 4L,M).

To complement these functional observations with a transcriptome‐level perspective on temporal differentiation dynamics, we analyzed bulk RNA‐seq data obtained at defined stages of vmDA differentiation (DIV 0, DIV 9, DIV 16, and DIV 35) under the optimized protocol. This analysis was designed to identify stage‐dependent transcriptional programs during vmDA differentiation. Protein‐coding genes were clustered using Mfuzz [19] to identify distinct temporal expression trajectories across differentiation stages. High‐confidence cluster members were defined based on membership scores, and the resulting eight temporal clusters were subsequently organized into three higher‐order transcriptional programs according to shared expression dynamics and similarity of functional enrichment profiles (Figure S6A,B). Group A comprised genes highly expressed at DIV 0 and progressively downregulated during differentiation and was enriched for ribosome biogenesis, mitochondrial gene expression and translation, and DNA replication‐related processes, consistent with exit from a pluripotent biosynthetic state (Figure S6C). Group B displayed transient activation during intermediate stages and was enriched for developmental patterning and morphogenetic processes, including dorsal/ventral pattern formation and regulation of nervous system development (Figure S6D). Notably, *EN1* was represented within this transitional program (cluster 5), consistent with acquisition of ventral midbrain competence prior to terminal neuronal maturation. In contrast, Group C exhibited progressive upregulation toward later stages and was enriched for gene sets related to synaptic transmission, neuronal excitability, regulated exocytosis, and dopaminergic synapse‐associated pathways (Tables S1–S4), defining a neuronal functional maturation program (Figure S6E). Together, these transcriptional programs illustrate a hierarchical organization of gene expression dynamics that parallels key stages of vmDA differentiation.

Finally, to assess the practical feasibility of implementing the optimized protocol in a cell therapy–oriented workflow, vmDA progenitors were cryopreserved at DIV 16 (Figure S7A). Post‐thaw analysis demonstrated that cell viability was maintained at approximately 80%, even after storage for up to one year (Figure S7B), indicating stability of the progenitor stage during long‐term preservation. Importantly, cryopreserved progenitors retained the capacity to differentiate into vmDA neurons in vitro, as evidenced by comparable expression of lineage markers compared with freshly differentiated controls (Figure S7C–H). These findings indicate that the optimized protocol supports scalable production and banking of vmDA progenitors without compromising their in vitro differentiation potential.

### 2.5 VmDA Neurons Generated by the Optimized Protocol Recapitulate PD‐associated α‐syn Pathology In Vitro

We next evaluated whether vmDA neurons generated using the optimized differentiation protocol could be applied to in vitro PD modeling. To this end, we employed a previously established optogenetics‐assisted α‐syn aggregation induction system (*OASIS*) [11], which enables rapid and controlled induction of pathogenic α‐syn aggregation in human neurons (Figure 5A). For these experiments, previously established PD patient‐derived hiPSC lines harboring either an *AAVS1::Mock* or *AAVS1::OASIS* knock‐in [11] (Figure 5B) were differentiated into vmDA progenitors and neurons using the optimized protocol (Figure 5C). Before inducing α‐syn aggregation, we first verified that the optimized differentiation protocol efficiently generated vmDA progenitors from PD hiPSCs, comparable to those derived from hESCs. Immunocytochemical analysis at DIV 16 demonstrated that vmDA progenitors derived from hESCs, *Mock* PD hiPSCs, and *OASIS* PD hiPSCs all exhibited comparably high proportions of EN1/LMX1A double‐positive cells (∼90%), with no statistically significant differences among the three groups (Figure S8A–C). These results indicate that the optimized differentiation protocol yields comparable vmDA progenitor specification from PD patient‐derived hiPSCs and hESCs.

**FIGURE 5:**
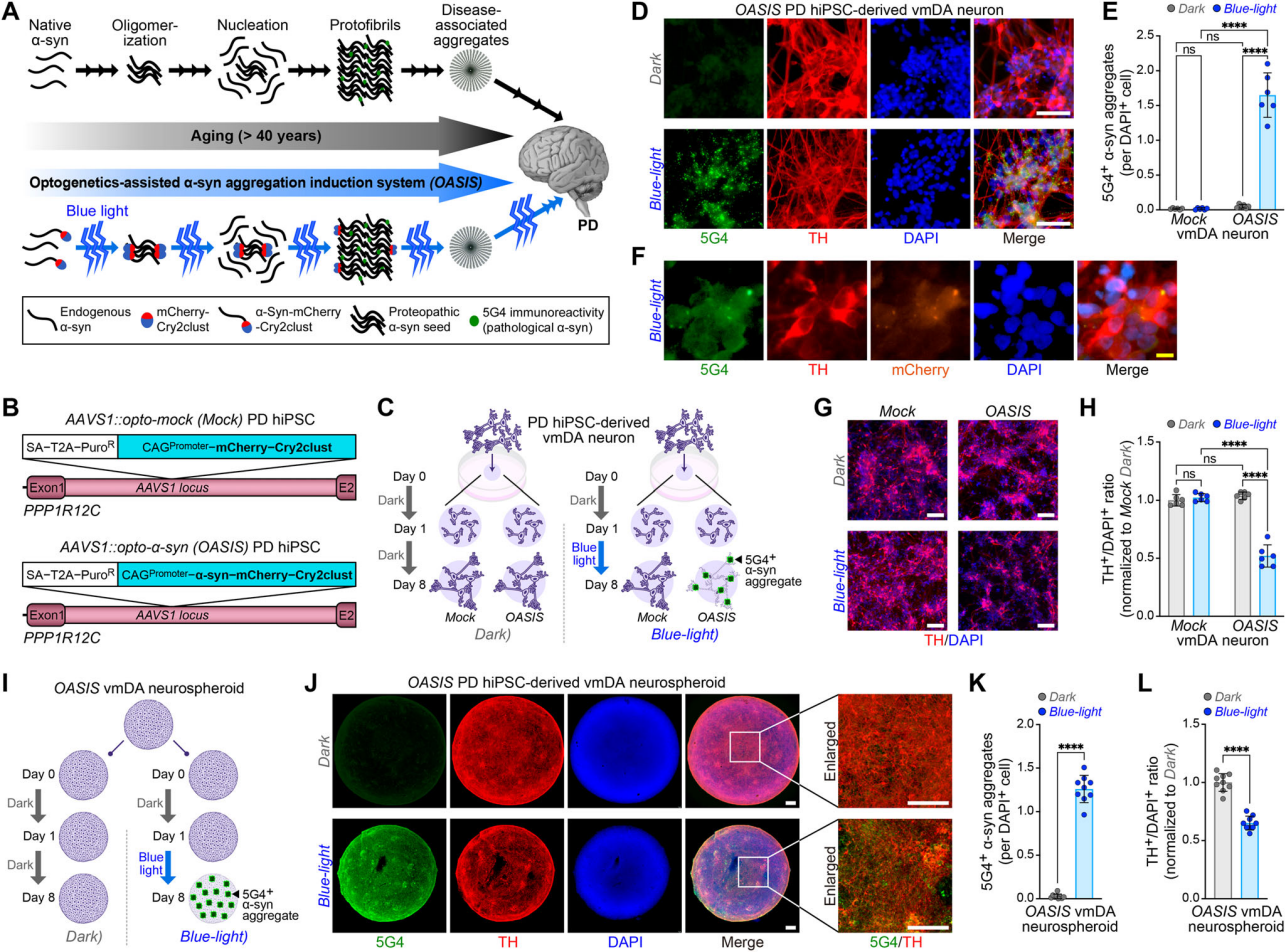
*OASIS*‐mediated induction of α‐synuclein aggregation selectively impaired vmDA neurons in vitro. (A) Schematic overview of age‐associated α‐synuclein (α‐syn) aggregation in PD and its optogenetics‐assisted acceleration using the *OASIS*. (B) Schematic representation of *AAVS1::opto‐mock* and *AAVS1::opto‐α‐syn* knock‐in alleles in PD hiPSCs. (C) Experimental timeline for *Mock* or *OASIS* PD hiPSC‐derived vmDA neurons seeded on DIV 26, followed by 24 h under dark conditions and subsequent 7‐day culture under dark conditions (*Dark*) or blue‐light illumination (*Blue‐light*; 14 µW/mm^2^ at 470 nm, 0.17 Hz, 0.5 s). (D–H) Representative immunofluorescence images of vmDA neurons stained for 5G4 (pathological α‐syn aggregates), TH, and mCherry (opto‐α‐syn) under *Dark* or *Blue‐light* conditions, with quantification of 5G4^+^ α‐syn aggregates per DAPI^+^ cell (E) and TH^+^/DAPI^+^ cell ratios (H) (*n* = 6 biological replicates). (I) Experimental timeline for DIV 26 *OASIS* PD hiPSC‐derived vmDA neurospheroids maintained under dark conditions for 24 h and subsequently cultured for 7 days under either *Dark* or *Blue‐light* conditions. (J) Representative optically cleared whole‐mount immunofluorescence images of *OASIS* vmDA neurospheroids cultured under *Dark* or *Blue‐light* conditions and stained for 5G4 and TH; enlarged views of the boxed regions are shown on the right. (K, L) Quantification of 5G4^+^ α‐syn aggregates per DAPI^+^ cell (K) and TH^+^/DAPI^+^ cell ratios (L) in *OASIS* vmDA neurospheroids (*n* = 9 biological replicates). Scale bars: 100 µm (white) and 10 µm (yellow). Data are presented as mean ± SD. Statistical significance was determined by two‐way ANOVA with Tukey's multiple‐comparison test (E, H) or two‐tailed unpaired Student's *t*‐test (K, L). ns, not significant; ^****^ *p* < 0.0001.

To induce α‐syn pathology, blue‐light illumination conditions were optimized for our experimental setup, and an intensity of 14 µW/mm^2^ was selected for subsequent experiments (Figure S8D–F). Under these conditions, optogenetic activation in *OASIS* vmDA neurons led to robust formation of pathogenic α‐syn aggregates, as evidenced by co‐localization of 5G4 [20]‐positive α‐syn aggregates with mCherry‐tagged α‐syn (Figure 5D–F). Induction of α‐syn aggregation resulted in a selective reduction of the TH‐positive neuronal population under blue‐light conditions compared with dark controls, indicating dopaminergic neuron‐specific vulnerability to α‐syn proteopathy (Figure 5G,H). These findings demonstrate that vmDA neurons derived from PD hiPSCs, when subjected to *OASIS*‐mediated induction of α‐syn aggregation, faithfully recapitulate a key pathological hallmark of PD—α‐syn aggregation‐associated degeneration of vmDA neurons—in a two‐dimensional (2D) culture system.

To further assess whether this pathological phenotype could be reproduced in a more tissue‐mimetic, organoid‐like environment, we next applied the *OASIS* system to three‐dimensional (3D) vmDA neurospheroids (Figure 5I and Figure S8G). Similar to observations in 2D cultures, blue‐light illumination in 3D vmDA neurospheroids induced robust formation of 5G4‐positive α‐syn aggregates that co‐localized with mCherry‐tagged α‐syn (Figure S8H). Notably, α‐syn aggregation in this 3D context also resulted in selective loss of TH‐positive neurons, confirming that dopaminergic neuron‐specific degeneration was preserved in an organoid‐like, tissue‐mimetic culture format (Figure 5J–L). Together, these results demonstrate that vmDA neurons generated by the optimized protocol, when subjected to *OASIS*‐mediated induction of α‐syn aggregation, reliably recapitulate key PD‐associated pathological features, including pathogenic α‐syn aggregation and selective degeneration of TH‐positive neurons, in both 2D and 3D in vitro systems.

### 2.6 Establishment of a Proteopathy‐based PD Mouse Model Enabling In Vivo Evaluation of vmDA Progenitors

To enable rigorous in vivo evaluation of vmDA progenitors generated by the optimized differentiation protocol under disease‐relevant conditions, we established a conditional proteopathy‐based PD mouse model in which intracellular amyloid stress is selectively induced in dopaminergic neurons. This model is based on regulated expression of the artificial amyloid‐forming peptide β23, which spontaneously assembles into β‐sheet‐rich aggregates immediately upon translation and has been shown to elicit proteotoxic stress and neurodegenerative phenotypes [21, 22]. A tetracycline‐responsive β23 expression cassette (TetP‐β23) was constructed and functionally validated in vitro using a Tet‐off configuration. β23 expression was tightly regulated by doxycycline (Dox) and resulted in the formation of punctate cytoplasmic aggregates accompanied by marked cytotoxicity, confirming effective inducible control of proteopathic stress (Figure S9A–D). Using a validated TetP‐β23 construct, two independent responder mouse lines were generated and crossed with DAT‐PF‐tTA driver mice to achieve dopaminergic neuron‐selective expression (Figure S9E). To avoid developmental toxicity, Dox was administered throughout gestation and early postnatal life, and β23 expression was induced by Dox withdrawal at 3 weeks of age (Figure S9F). Immunofluorescence analysis confirmed robust β23 aggregate formation selectively within dopaminergic neurons of the ventral midbrain (Figure S9G,H).

Following β23 induction, DAT‐PF‐tTA; TetP‐β23 (β23 Tg) mice exhibited progressive degeneration of the nigrostriatal dopaminergic system. Quantitative analyses revealed a substantial reduction of TH‐positive neurons in the SNpc by 8 weeks of age, with further loss observed at 14 weeks, whereas littermate controls remained unaffected (Figure S10A–C). In parallel with the loss of TH‐positive neurons in the SNpc, TH‐positive dopaminergic fibers in the striatum were progressively depleted over time (Figure S10D,E). Behavioral assessment demonstrated worsening motor impairment in β23 Tg mice, as measured by both pole and rotarod tests, whereas control animals maintained stable motor performance (Figure S10F,G). Prior to doxycycline withdrawal, β23 Tg mice did not exhibit detectable neuropathological changes or motor deficits and were indistinguishable from DAT‐PF‐tTA control mice. Together, these features establish a stringent proteopathy‐associated in vivo PD context that enables systematic evaluation of the survival, differentiation, and functional impact of transplanted vmDA progenitors, providing the foundation for subsequent transplantation studies.

### 2.7 vmDA Progenitor Transplantation Rescues Motor Deficits in a Proteopathy‐based PD Mouse Model

On the basis of the progressive nigrostriatal degeneration and sustained motor deficits observed in β23 Tg mice, this model was used as a proteopathy‐based PD mouse model to assess the therapeutic efficacy of vmDA progenitor transplantation. Using this model, vmDA progenitors differentiated to DIV 16 using the optimized protocol were bilaterally transplanted into the striatum of 14‐week‐old mice, an advanced PD stage characterized by extensive nigrostriatal degeneration and stable motor impairment (Figure 6A). Motor performance was assessed prior to transplantation and at 4 and 8 weeks post‐transplantation according to the experimental timeline, and the corresponding behavioral outcomes are presented in Figure 6B. Before grafting, PD mice exhibited pronounced bradykinesia and impaired motor coordination compared with control animals. In PBS‐injected PD mice, motor performance remained largely unchanged at both post‐transplantation time points. In contrast, vmDA‐transplanted PD mice showed a robust and sustained rescue of motor deficits. In the pole test, vmDA‐transplanted PD mice exhibited descent latencies that were indistinguishable from those of control animals at both 4 and 8 weeks post‐transplantation, indicating near‐complete rescue of bradykinesia. In the rotarod test, vmDA‐transplanted PD mice showed a marked improvement in motor coordination relative to PBS‐injected PD mice at both time points (Figure 6B).

**FIGURE 6:**
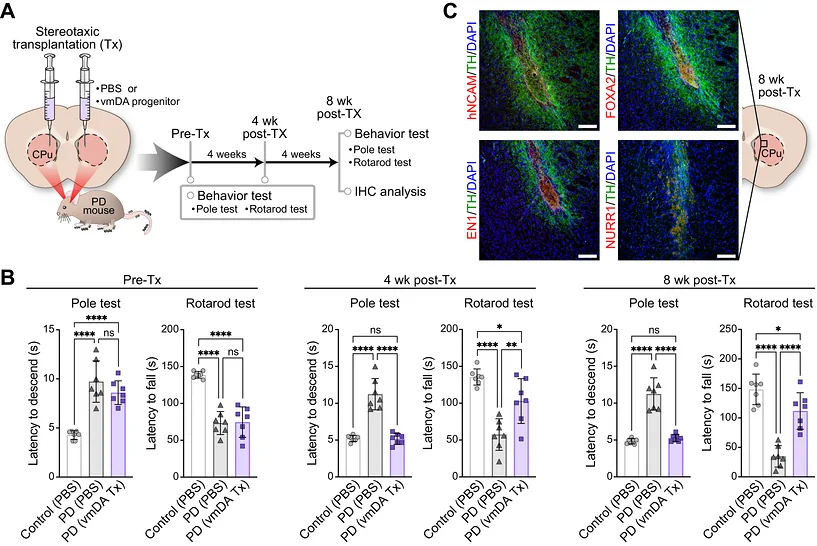
Transplantation of vmDA progenitors rescued motor deficits in an advanced PD mouse model. (A) Schematic of the experimental paradigm. hESC‐derived vmDA progenitors differentiated to DIV 16 using the optimized protocol were bilaterally transplanted into the striatum of 14‐week‐old DAT‐PF‐tTA;TetP‐β23 mice, an advanced proteopathy‐based PD mouse model, followed by behavioral assessment at baseline (pre‐transplantation; pre‐Tx) and at 4 and 8 weeks post‐Tx, with immunohistochemical analysis performed at 8 weeks post‐Tx. (B) Motor function assessment of littermate DAT‐PF‐tTA control mice injected with PBS (white bars), DAT‐PF‐tTA;TetP‐β23 PD mice injected with PBS (grey bars), and PD mice receiving vmDA progenitor Tx (purple bars) (*n* = 7 mice). Behavioral tests were performed at pre‐Tx and at 4 and 8 weeks post‐Tx. Bradykinesia was assessed using the pole test, and motor coordination using the rotarod test. (C) Representative immunofluorescence images showing survival and differentiation of grafted vmDA progenitors in the striatum at 8 weeks post‐Tx. Graft‐derived cells were identified by hNCAM expression, and vmDA lineage markers (FOXA2, EN1, NURR1, and TH) were detected in grafted cells. Nuclei were counterstained with DAPI. Scale bars, 100 µm. Data are presented as mean ± SD. Statistical significance was determined by one‐way ANOVA with Tukey's multiple‐comparison test. ns, not significant; ^*^ *p* < 0.05, ^**^ *p* < 0.01, ^****^ *p* < 0.0001.

To determine whether the observed behavioral rescue was accompanied by graft survival and dopaminergic differentiation, histological analyses were performed at 8 weeks post‐transplantation. hNCAM‐positive graft‐derived cells were readily detected within the striatum, confirming survival of transplanted human cells (Figure 6C and Figure S11A–D). TH‐positive cell bodies were observed within hNCAM‐positive graft regions, and their numbers within the graft area were quantified for each mouse (Figure S11E). A substantial proportion of graft‐derived cells expressed TH and co‐expressed established vmDA lineage markers, including EN1, FOXA2, and NURR1, indicating differentiation toward a vmDA neuronal phenotype in vivo (Figure 6C and Figure S11F). Together, these results demonstrate that vmDA progenitors generated by the optimized differentiation protocol can survive and differentiate within a proteopathy‐associated PD brain environment and effectively rescue motor deficits in a severe and progressive genetic model of dopaminergic neurodegeneration.

## 3 Discussion

In this study, we established a developmentally guided differentiation protocol that enables efficient and reproducible generation of vmDA lineage cells from hPSCs. By temporally optimizing SHH, WNT/β‐catenin, and FGF8 signaling, the protocol robustly specifies vmDA progenitors co‐expressing EN1, OTX2, LMX1A, and FOXA2 and supports their maturation into molecularly and functionally mature vmDA neurons. The protocol also preserves practical translational features, including cryopreservation of DIV 16 progenitors with maintained post‐thaw viability and differentiation capacity.

Alternative strategies have used distinct sources of information to optimize vmDA cell products. Kirkeby et al. [23] correlated progenitor‐stage gene‐expression profiles from more than 30 transplanted hESC‐derived batches with graft outcomes, identified caudal ventral midbrain markers predictive of high in vivo dopaminergic yield, and used these predictors to refine FGF8 patterning in a GMP‐adapted process. More recently, Zhang et al. [24] developed a full‐term 3D, xeno‐free differentiation system and combined single‐cell and spatial transcriptomic analyses of grafts with clonal lineage tracing to identify the donor origins of desired and off‐target graft populations. This information was then used to refine the differentiation process and reduce off‐target lineages. Accordingly, Kirkeby et al. provide progenitor‐stage predictors of graft performance, whereas Zhang et al. use lineage‐resolved graft analysis to identify and reduce off‐target populations. By contrast, optimization in the present study was guided by stage‐specific molecular readouts obtained during in vitro differentiation, yielding a highly marker‐concordant DIV 16 progenitor population that formed the basis for subsequent 3D maturation and evaluation in complementary in vitro and in vivo proteopathy‐based PD models.

At the protocol level, established vmDA differentiation methods use related developmental cues but implement them through different combinations, concentrations, and treatment windows. Kim et al. [15] promoted EN1 induction without exogenous FGF8 through early biphasic activation of WNT signaling within a protocol incorporating dual‐SMAD inhibition and early SHH exposure. Nishimura et al. [25] activated the SHH pathway with purmorphamine rather than exogenous SHH protein and incorporated WNT5A‐mediated noncanonical WNT activation into a chemically defined adherent differentiation protocol. In the present study, the concentrations and treatment windows for SHH/PMP, CHIR, and FGF8 were systematically evaluated to establish the optimized protocol (Figures 2 and 3 and Figures S1–S3). The resulting feeder‐free, KSR‐free protocol combines exogenous SHH with PMP from DIV 1 to DIV 16, CHIR administered at 0.8 µm beginning on DIV 0 and increased to 7 µm on DIV 11 through DIV 16, and FGF8 from DIV 9 to DIV 16. Maintaining SHH/PMP exposure and the high‐dose CHIR phase through DIV 16 increased EN1 and LMX1A specification (Figures 2 and 3). At DIV 16, the proportions of OTX2/EN1, LMX1A/FOXA2, LMX1A/EN1, and FOXA2/EN1 double‐positive cells ranged from approximately 87% to 94% (Figure 4C,D). EN1 is a central determinant of ventral midbrain identity and contributes to the development and long‐term maintenance of SNpc dopaminergic neurons [18]. The high level of EN1 co‐expression with OTX2, LMX1A, and FOXA2 therefore provides strong evidence of coordinated regional and floor‐plate specification within the progenitor population. Maintenance of EN1 together with other vmDA markers during neuronal differentiation and after transplantation further supports continuity of vmDA lineage identity across the downstream contexts examined. Consistent with these findings, direct comparison with the Kim et al. protocol showed higher *EN1* and *LMX1A* expression at DIV 16, while *FOXA2* and *TH* expression was comparable, and higher *EN1*, *LMX1A*, *FOXA2*, and *TH* expression at DIV 40 (Figure S5). Together, these findings establish the reproducible generation of highly enriched EN1‐positive DIV 16 progenitors with coordinated vmDA identity as the principal technical advance of the optimized protocol. The protocol further incorporates a neurosphere‐based maturation phase after vmDA progenitor specification. During protocol development, a 10‐day 3D neurosphere phase increased the proportion of TH‐positive cells from approximately 46% to 80% and TUJ1‐positive cells from approximately 81% to 95% relative to continuous 2D maturation (Figure 1). This efficiency‐enhancing step was incorporated into the final workflow from DIV 16 to DIV 26 and supported the subsequent generation of molecularly and functionally mature vmDA neurons. Thus, the optimized protocol integrates the reproducible generation of highly enriched EN1‐positive vmDA progenitors with efficient 3D neuronal maturation.

In addition to marker‐level validation, transcriptome‐level analysis revealed a hierarchical organization of transcriptional programs that parallels key stages of vmDA differentiation. An early reduction of pluripotency‐associated biosynthetic processes was followed by activation of a developmental patterning module that included *EN1*, consistent with acquisition of ventral midbrain competence prior to terminal neuronal maturation. This transitional phase was accompanied by activation of a late transcriptional module enriched for synaptic transmission, membrane excitability, and dopaminergic synapse‐associated pathways. Collectively, these findings provide a systems‐level framework that supports the developmental fidelity of the optimized vmDA differentiation strategy.

To functionally validate the utility of hPSC‐derived vmDA lineage cells, including both progenitors and neurons, we applied complementary proteopathy‐based PD modeling strategies. In vitro, optogenetic induction of α‐syn aggregation using *OASIS* [11] elicited robust pathogenic aggregate formation and selective degeneration of TH‐positive neurons in both 2D cultures and 3D neurospheroids, demonstrating that vmDA neurons generated by the protocol exhibit proteopathy‐associated vulnerability. In vivo, we established a conditional β23 Tg model that produces progressive, dopaminergic neuron‐selective nigrostriatal degeneration and sustained motor impairment within a defined timeframe. Transplantation of hESC‐derived vmDA progenitors into this stringent disease environment resulted in graft survival, in vivo differentiation into TH‐positive neurons co‐expressing EN1, FOXA2, and NURR1, and marked improvement in motor behavior. Notably, behavioral recovery differed between assays, with pole test performance approaching control levels and substantial, albeit incomplete, improvement in the rotarod test, likely reflecting distinct motor domains assessed by each task [26, 27, 28]. The magnitude of behavioral improvement observed in this severe proteopathy‐based model contrasts with more modest outcomes often reported in acute lesion paradigms [1]. One plausible explanation is that the β23‐based conditional PD model provides a stable, dopaminergic neuron–selective, and progressive pathological milieu without the acute cytotoxic and inflammatory effects associated with neurotoxin‐based models [29, 30]. This environment may permit sustained survival and maturation of transplanted progenitors, ultimately leading to functional improvement of the selectively impaired nigrostriatal motor pathway in vivo. In addition, the high degree of vmDA progenitor specification achieved prior to transplantation may favor differentiation trajectories compatible with functional dopaminergic integration.

While this study establishes robust lineage fidelity and functional benefit, certain aspects remain to be further explored. Our transcriptome analysis was performed using bulk RNA‐seq across defined differentiation stages. This approach was appropriate for identifying stage‐dependent transcriptional programs during vmDA differentiation, but it does not resolve cellular heterogeneity, rare cell populations, or cell‐state diversity at single‐cell resolution. Future studies incorporating scRNA‐seq or snRNA‐seq would provide a more detailed understanding of cellular composition, maturation trajectories, and subtype‐related heterogeneity within the differentiated cultures. Although GIRK2 was broadly co‐expressed with TH in vitro, GIRK2 expression alone is insufficient to establish A9 subtype identity. Likewise, the graft analysis showed vmDA‐lineage marker expression in representative TH‐positive cells but did not include a broader panel of subtype‐associated markers. Accordingly, the present data support a vmDA neuronal phenotype in vitro and dopaminergic differentiation with vmDA‐lineage features after transplantation, whereas definitive A9 subtype assignment will require additional subtype‐associated marker analysis. In addition, the molecular pathways linking β23 aggregate formation to dopaminergic neurodegeneration remain to be further elucidated, and the present analysis focuses on graft survival, lineage‐specific differentiation, and behavioral outcomes rather than on detailed assessment of graft‐derived synaptic integration, target reinnervation, or circuit‐level remodeling. Future studies integrating mechanistic profiling with quantitative connectivity analyses will help refine the relevance of the β23‐based PD model and clarify how developmentally specified vmDA progenitors confer functional benefit in vivo.

Overall, these findings support a framework in which stringent, developmentally guided vmDA specification gives rise to vmDA lineage cells whose functional utility is demonstrated through in vitro proteopathy modeling and in vivo transplantation in a progressive neurodegeneration model, providing a robust foundation for PD modeling and cell therapy.

## 4 Conclusion

In summary, we developed a developmentally guided differentiation strategy that enables efficient and reproducible generation of vmDA progenitors and neurons with high molecular fidelity. By optimizing SHH, WNT/β‐catenin, and FGF8 signaling in a temporally defined manner, the protocol robustly induces EN1‐positive vmDA progenitors that mature into functionally competent dopaminergic neurons. Using the *OASIS* platform, these vmDA neurons recapitulated α‐syn aggregation–associated degeneration in both 2D and 3D formats, supporting their utility for proteopathy‐based PD modeling. In parallel, we established a conditional proteopathy‐based PD mouse model that exhibits progressive, dopaminergic neuron–selective nigrostriatal degeneration and sustained motor impairment. Transplantation of hESC‐derived vmDA progenitors into this stringent disease environment resulted in graft survival, in vivo vmDA neuronal differentiation, and significant rescue of motor deficits. Collectively, these findings establish the optimized differentiation protocol and complementary proteopathy‐based modeling framework as a robust platform for PD modeling and cell therapy.

## 5 Experimental Section

### 5.1 Cell Culture

Undifferentiated H9 hESCs (WA09; WiCell) were maintained on mitotically inactivated mouse embryonic fibroblast (MEF) feeder layers (Gibco, A34181). *Mock* (*AAVS1::opto‐mock*) and *OASIS* (*AAVS1::opto‐α‐syn*) knock‐in derivatives of the *SNCA* triplication PD hiPSC line (ND27760‐8; 46, XX) [11] were maintained under the same conditions. Cells were cultured in hPSC medium consisting of DMEM/F12 (Gibco, 11330032) supplemented with 20% KnockOut serum replacement (KSR; Gibco, 10828028), 0.1 mm MEM non‐essential amino acids (MEM‐NEAA; Gibco, 11140050), 2 mm GlutaMAX (Gibco, 35050079), 55 µm β‐mercaptoethanol (Gibco, 21985023), and 10 ng/mL fibroblast growth factor‐2 (FGF2; R&D Systems, 4114‐TC‐01M), as previously described [31]. Cells were incubated at 37°C in a humidified atmosphere containing 5% CO_2_, with daily medium changes. For feeder‐free hPSC culture, colony fragments mechanically picked from hPSCs cultured on MEF feeder layers were transferred onto iMatrix‐511 Silk (iMatrix; Matrixome, MX892021)‐coated plates and cultured in StemFit Basic04 CT medium (StemFit medium; Ajinomoto, AJBASIC04CT) supplemented with 10 µm ROCK inhibitor (Y‐27632; Tocris Bioscience, 1254). After 24 h, the medium was replaced with fresh StemFit medium. Cells were routinely passaged every 7 days under feeder‐free conditions. All hESC experiments were determined to be exempt from review by the Institutional Review Board of Hanyang University (exemption number HYUIRB‐202512‐002). All experiments were performed using hPSCs at passages 30–55.

HEK293T cells were maintained in DMEM (Gibco, 119950) supplemented with 10% fetal bovine serum (FBS; Gibco, 26140079) and 1% penicillin/streptomycin (P/S; Gibco, 15140122) in a humidified atmosphere containing 5% CO_2_ at 37°C. Transient transfections were performed using X‐tremeGENE HP DNA Transfection Reagent (Roche, 6366244001) according to the manufacturer's instructions.

### 5.2 Generation of the TH::TH‐T2A‐eGFP (TH::eGFP) Knock‐in hESC Line

Feeder‐free H9 hESCs (passage 37) were dissociated into single cells using Accutase (Thermo Fisher Scientific, A6964). A total of 1 × 10^6^ cells were resuspended in Opti‐MEM I Reduced Serum Medium (Gibco, 31985062) containing 2 µg of hCas9 plasmid (Addgene, plasmid #41815; a gift from Dr. George Church), 6 µg of donor plasmid (Donor‐T1; a gift from Dr. Masato Koike), and 2 µg of gRNA plasmid (sgRNA‐T1; a gift from Dr. Masato Koike). Cells were electroporated at 125 V for 5 ms using a NEPA21 Super Electroporator (Nepa Gene) according to the manufacturer's instructions. Immediately after electroporation, cells were plated onto mitotically inactivated MEF feeder layers in hPSC medium supplemented with 10 µm Y‐27632. Four days post‐electroporation, puromycin selection was initiated by adding 0.5 µg/mL puromycin (InvivoGen, ant‐pr‐1) to the hPSC medium. After 4 days of selection, cells were cultured for an additional 5 days in puromycin‐free hPSC medium to allow recovery. Puromycin‐resistant colonies were individually picked at 13 days post‐seeding under a stereomicroscope and expanded for further analysis. Genomic DNA was extracted from each hESC colony using the DNeasy Blood & Tissue Kit (Qiagen, 69504) according to the manufacturer's protocol. PCR‐based genotyping was performed to confirm correct knock‐in integration following a previously reported strategy [32]. After validation of TH expression–reporting capability, a single representative *TH::eGFP* knock‐in clone was selected based on correct genomic integration and robust reporter fidelity and used for subsequent vmDA neuronal differentiation experiments.

### 5.3 vmDA Neuronal Differentiation

For vmDA neuronal differentiation, hPSCs were cultured for 8 days on iMatrix‐coated plates in StemFit medium and then dissociated into single cells using Accutase. Cells were then seeded at a density of 130 000–160 000 cells/cm^2^ onto fresh iMatrix‐coated plates in StemFit medium supplemented with 10 µm Y‐27632. Differentiation was initiated after 3–4 days, when cells reached 95–100% confluence. To induce vmDA neuronal differentiation, confluent hPSC cultures (DIV 0) were treated with 100 nm LDN193189 (LDN; STEMCELL Technologies, 72147), 10 µm SB431542 (SB; Stemgent, 04‐0010‐10), and 0.8 µm CHIR99021 (CHIR; Tocris Bioscience, 4423) in Neurobasal Medium (Gibco, 21103049) supplemented with 2 mm GlutaMAX and 1× B‐27 Supplement (Gibco, 12587010) (B27 medium). From the next day (DIV 1), cells were treated with 2 µm purmorphamine (PMP; Cayman Chemical, 10009634) and 100 ng/mL recombinant human sonic hedgehog C25II (SHH; PeproTech, 100–45), and SB was withdrawn on DIV 5. Starting on DIV 9, the medium was supplemented with 100 ng/mL recombinant human FGF‐8b (FGF8; PeproTech, 100–25). On DIV 11, the concentration of CHIR was increased to 7 µm, and differentiation was allowed to proceed until DIV 16. On DIV 16, cells were dissociated into single cells using Accutase, and passaged at a density of 150 000–200 000 cells per well into ultra‐low attachment 96‐well plates (Corning, 7007) in the B27 medium supplemented with 20 ng/mL recombinant human brain‐derived neurotrophic factor (BDNF; PeproTech, 450‐02), 20 ng/mL recombinant human glial cell line‐derived neurotrophic factor (GDNF; PeproTech, 450‐10), 200 µm ascorbic acid (AA; Sigma–Aldrich, A4034), 500 µm dibutyryl cyclic AMP (dbcAMP; Sigma–Aldrich, D0627), 10 µm DAPT (TCI, D4257), 1 ng/mL recombinant human transforming growth factor‐beta 3 (TGFβ3; R&D Systems, 243‐B3‐200), 10 µm Y‐27632, and 1% P/S (Gibco, 15140122). Y‐27632 was removed the next day (DIV 17), and the medium was subsequently changed every other day for 10 days. On the final day of the 10‐day neurosphere culture period (DIV 26), cells were dissociated into single cells using Accutase and plated as 10 µL droplets (15 000–20 000 cells/µL) at the center of dishes coated with poly‐L‐ornithine hydrobromide (PO; Sigma–Aldrich, P3655), laminin (Sigma–Aldrich, L2020), and fibronectin (Corning, 356008). These cells were cultured in the B27 medium supplemented with 20 ng/mL BDNF, 20 ng/mL GDNF, 200 µm AA, 500 µm dbcAMP, 10 µm DAPT, 1 ng/mL TGFβ3, 10 µm Y‐27632, and P/S. Y‐27632 was removed the following day (DIV 27), and the medium was changed every two days until differentiation was completed.

### 5.4 Flow Cytometry

Cells were dissociated mechanically following treatment with Accutase and 40 µg/mL DNase I (Roche, 11284932001) for 10–15 min at 37°C. Dissociated cells were resuspended in FACS buffer consisting of PBS (Corning, 21‐031‐CV) supplemented with 1% FBS (Gibco, 16000044), 9% DMEM/F12, and 40 µg/mL DNase I. Flow cytometry was performed on a FACSCanto II (BD Biosciences). Data were analyzed using FlowJo v10 (BD Biosciences).

### 5.5 Blue‐Light Stimulation and Culture Conditions

Blue‐light stimulation (14 µW/mm^2^ at 470 nm, 0.17 Hz, 0.5 s pulses; unless otherwise indicated) was applied using a customized TouchBright W‐96 LED excitation system (TB‐X6‐Z004; Live Cell Instrument) placed inside the incubator. Illumination parameters for each well were controlled via Live Cell Instrument software on a desktop computer located outside the incubator. For phototoxicity assays, stimulation was applied for 7 days at intensities ranging from 3.7 to 29.6 µW/mm^2^ at 470 nm (0.17 Hz, 0.5 s pulses) using a 96‐well black plate (SPL Life Sciences, 33296) to ensure selective illumination. The actual light intensity at the culture surface was measured with a LaserCheck power meter (Coherent). In subsequent experiments, blue light was delivered for 7 days at 14 µW/mm^2^ at 470 nm (0.17 Hz, 0.5 s pulses). To minimize nonspecific optogenetic α‐syn aggregate formation caused by ambient light, cultures were maintained in a light‐controlled environment, and illumination was restricted to red light.

### 5.6 Cell Viability Assay

Because *OASIS* knock‐in vmDA neurons undergo cell death upon blue‐light stimulation due to optogenetic induction of α‐syn aggregation [11], we performed a parallel assay to evaluate whether additional phototoxicity was caused by light exposure itself. *Mock* and *OASIS* knock‐in vmDA neurons were seeded as 10 µL droplets containing 150 000 cells into 96‐well black plates (SPL Life Sciences, 33296) and subjected to 7 days of blue‐light stimulation at intensities ranging from 3.7 to 29.6 µW/mm^2^ at 470 nm (0.17 Hz, 0.5 s pulses). Cell viability was assessed using the CCK‐8 Cell Counting Kit (ABBKINE, KTA1020) according to the manufacturer's instructions.

For the trypan blue exclusion assay, HEK293T cells were seeded onto 12‐well plates at a density of 1 × 10^5^ cells per well. Seeded cells were transfected with designated constructs and cultured in DMEM medium supplemented with 10% FBS. For doxycycline‐treated groups, cells were additionally treated with doxycycline at a final concentration of 100 ng/mL. After 24–48 h, the cells were harvested using trypsinization, washed with 1× PBS twice, and resuspended in 1× PBS. The cell suspension was then mixed with 0.4% trypan blue (w/v) in equal proportions and incubated for 1 min at room temperature. Live and dead cells were quantified using a Countess II FL Cell Counter (Life Technologies).

### 5.7 RNA Sequencing and Transcriptome Analysis

Total RNA was extracted from hESC‐derived vmDA lineage cells collected at DIV 0, DIV 9, DIV 16, and DIV 35 during differentiation using the optimized protocol, with two biological replicates per time point, using TRIzol Reagent (Invitrogen, 15596026). RNA‐seq libraries were prepared using the NEBNext Ultra II RNA Library Prep Kit for Illumina (New England Biolabs, E7770) and sequenced on an Illumina NovaSeq X Plus platform (Illumina) to generate paired‐end 150‐bp reads. Raw FASTQ files were aligned to the human reference genome (GRCh38.p14) using STAR (v2.7.10a) [33] and Samtools (v1.17) [34]. Gene‐level read counts were obtained using the featureCounts [35] function from the Rsubread (v2.16.1). Count data were normalized to fragments per kilobase of transcript per million mapped reads (FPKM) using the rpkm function from edgeR (v3.42.4) [36] (Table S5).

After averaging gene expression values across biological replicates at each time point, temporal expression patterns were analyzed using Mfuzz (v2.62.0) [19] to cluster genes across differentiation stages (c = 8). Only protein‐coding genes annotated in the CCDS database [37] were included in the analysis (18 307 genes). Genes within each cluster were filtered using a minimum membership score (min.acore = 0.5) based on the acore function in Mfuzz, and the complete gene lists for all clusters (6 295 genes) are provided in Table S6. To facilitate higher‐level interpretation, the eight Mfuzz clusters were further grouped into three transcriptional programs (Groups A–C) according to shared temporal expression dynamics and similarity of functional enrichment profiles.

Functional over‐representation analysis (ORA) was performed using the clusterProfiler package (v4.16.0) [38]. The org.Hs.eg.db and GO.db annotation databases (v3.21.0) were used for Gene Ontology (GO; biological process, molecular function, and cellular component) analysis. KEGG pathways, MSigDB gene sets, and Reactome pathways were analyzed using clusterProfiler and ReactomePA (v1.52.0). Enrichment was assessed using the hypergeometric test with Benjamini–Hochberg multiple testing correction, and terms with adjusted *p* values (FDR) < 0.05 were considered significant. For visualization, the top 10 enriched GO biological process terms for each group were selected based on adjusted *p* values. Dot plots were generated using ggplot2 (v4.0.0), where the x‐axis represents −log_10_(adjusted *p* value), point size indicates the number of genes contributing to each term (Count), and color indicates fold enrichment relative to the background. Full enrichment results are provided in the corresponding supplementary tables (Tables S1–S4).

### 5.8 Reverse Transcription Quantitative PCR (RT‐qPCR) Analysis

Total RNA was extracted from cultured cells using TRIzol Reagent (Invitrogen, 15596026), and cDNA was synthesized using ReverTra Ace qPCR RT Master Mix with gDNA Remover (Toyobo, FSQ‐301) according to the manufacturer's instructions. RT‐qPCR was performed using THUNDERBIRD Next SYBR qPCR Mix (Toyobo, QPX‐201) on a CFX Connect Real‐Time PCR Detection System (Bio‐Rad). Expression levels of target genes were normalized to *GAPDH* expression. All primers used for RT‐qPCR are listed in Table 1.

**TABLE 1:** Sequences of primers used for RT‐qPCR and genotyping.

Genes	Forward	Reverse
*DAT^tTA^*	5'‐AGAAGAAGGAAACAGACTTCCTC‐3'	5'‐GCTTGTTCTTCACGTGCCAGT‐3'
*DAT^WT^*	5'‐AGAAGAAGGAAACAGACTTCCTC‐3'	5'‐TGATGAGGGTGGAGTTGGTC‐3'
*EN1*	5'‐CTGGGTGTACTGCACACGTT‐3'	5'‐GCTTGTCCTCCTTCTCGTTC‐3'
*FOXA2*	5'‐CCGTTCTCCATCAACAACCT‐3'	5'‐GGGGTAGTGCATCACCTGTT‐3'
*GAPDH*	5'‐AGGGCTGCTTTTAACTCTGGT‐3'	5'‐CCCCACTTGATTTTGGAGGGA‐3'
*Gapdh*	5'‐TGGGTGGAGTGTCCTTTATCC‐3'	5'‐TATGCCCGAGGACAATAAGG‐3'
*GBX2*	5'‐GTTCCCGCCGTCGCTGATGAT‐3'	5'‐GCCGGTGTAGACGAAATGGCCG‐3'
*KROX20*	5'‐TTGACCAGATGAACGGAGTG‐3'	5'‐CTTGCCCATGTAAGTGAAGGT‐3'
*LMX1A*	5'‐CAGCAGCAAGATCAGCAGAA‐3'	5'‐AGGGGTTCATGATTCCTTCC‐3'
*NURR1*	5'‐CGCTTCTCAGAGCTACAGTTAC‐3'	5'‐TGGTGAGGTCCATGCTAAAC‐3'
*OTX2*	5'‐ACAAGTGGCCAATTCACTCC‐3'	5'‐GAGGTGGACAAGGGATCTGA‐3'
*TetP‐β23*	5'‐TGCTCCATTTTGCGTGACTCT‐3'	5'‐TCTCGATCTCGATGTCGCCG‐3'
*TH*	5'‐TCATGTCCCCGCGGTTCATT‐3'	5'‐TCCCCTCCTTCTCCTCAAAGG‐3'

### 5.9 Western Blot Analysis

Cells were collected, washed twice with PBS, and lysed in RIPA buffer (150 mm NaCl, 50 mm Tris [pH 8.0], 1% NP‐40, 1% SDS, and 0.5% sodium deoxycholate) supplemented with a protease inhibitor cocktail (Xpert Protease Inhibitor Cocktail Solution; GenDEPOT, P3100‐001). After sonication, the samples were clarified by centrifugation at 15 000 × *g* for 15 min at 14°C. Equal amounts of protein (15 µg) were boiled at 98°C for 2 min in Laemmli sample buffer (Bio‐Rad, 1610737), supplemented with β‐mercaptoethanol (Bio‐Rad, 1610710), resolved by SDS–PAGE, and transferred onto nitrocellulose membranes (GE Healthcare, 10600001). Membranes were blocked with 5% skim milk (BD Difco, 232100) in TBST (TBS with 0.1% Tween 20; Cell Signaling Technology, 9997) for 1 h at room temperature and washed three times with TBST for 10 min each. Membranes were incubated overnight with primary antibodies at 4°C, washed three times with TBST, and incubated with HRP‐conjugated secondary antibodies for 1 h at room temperature. After washing three times with TBST, signals were detected using an ECL substrate (Thermo Scientific, 34577). Western blot analyses were performed with the indicated antibodies (Table 2).

**TABLE 2:** Details of antibodies used in this study.

Primary antibodies					
anti‐	Company	Cat. No.	Isotype	Application	Dilution
Aggregated α‐syn, clone 5G4 (5G4)	Millipore	MABN389	Mouse IgG_1_	Immunocytochemistry	1:1,000
DAT	Millipore	MAB369	Rat IgG_2a_	Immunocytochemistry	1:500
EN1	DSHB	4G11	Mouse IgG_1_	Immunocytochemistry	1:50
				Immunohistochemistry	1:50
FLAG	Sigma–Aldrich	F1804	Mouse IgG_1_	Immunocytochemistry	1:1,000
				Western Blot	1:5,000
FOXA2	R&D systems	AF2400	Goat IgG	Immunocytochemistry	1:1,000
				Immunohistochemistry	1:1,000
FOXA2	Santa Cruz Biotechnology	sc‐374376	Mouse IgG_1_	Immunocytochemistry	1:1,000
GIRK2	Alomone	APC‐006	Rabbit IgG	Immunocytochemistry	1:500
HA	Invitrogen	PA1‐985	Rabbit IgG	Immunohistochemistry	1:1,000
hNCAM	Santa Cruz Biotechnology	sc‐106	Mouse IgG_1_	Immunohistochemistry	1:500
LMX1A	abcam	ab139726	Rabbit IgG	Immunocytochemistry	1:1,000
MAP2	Sigma–Aldrich	M1406	Mouse IgG_1_	Immunocytochemistry	1:500
mCherry	Invitrogen	M11217	Rat IgG_2a_	Immunocytochemistry	1:1,000
NESTIN	Biolegend	656802	Mouse IgG_1_	Immunocytochemistry	1:1,000
NURR1	R&D Systems	PP‐N1404‐00	Mouse IgG_2a_	Immunocytochemistry	1:500
				Immunohistochemistry	1:500
OTX2	R&D Systems	AF1979	Goat IgG	Immunocytochemistry	1:1,000
PAX6	Biolegend	901301	Rabbit IgG	Immunocytochemistry	1:1,000
PITX3	Millipore	AB5722	Rabbit IgG	Immunocytochemistry	1:200
PSD95	NeuroMab	75‐028	Mouse IgG_2a_	Immunocytochemistry	1:500
SYN1	Synaptic Systems	106 103	Rabbit IgG	Immunocytochemistry	1:500
TH	ImmunoStar	22941	Mouse IgG_1_	Immunocytochemistry	1:1,000
TH	Novus Biologicals	NB300‐109	Rabbit IgG	Immunohistochemistry	1:5,000
TH	Pel‐Freez Biologicals	P40101‐150	Rabbit IgG	Immunocytochemistry	1:1,000
				Immunohistochemistry	1:500
TUJ1	Covance	PRB‐435P	Rabbit IgG	Immunocytochemistry	1:1,000
TUJ1	Biolegend	801201	Mouse IgG_2a_	Immunocytochemistry	1:1,000

HRP‐conjugated primary antibody						
anti‐	Company	Cat. No.	Isotype	Conjugate	Application	Dilution
β‐Actin	Sigma–Aldrich	A3854	Mouse IgG_1_	HRP	Western Blot	1:5,000

Secondary antibodies						
anti‐	Company	Cat. No.	Host	Conjugate	Application	Dilution
Goat IgG	Invitrogen	A‐32814	Donkey	Alexa Fluor Plus 488	Immunocytochemistry	1:1,000
Goat IgG	Invitrogen	A‐32849	Donkey	Alexa Fluor Plus 647	Immunocytochemistry	1:1,000
					Immunohistochemistry	1:1,000
Mouse IgG	GeneTex	GTX213111‐01	Goat	HRP	Western Blot	1:1,000
Mouse IgG	Invitrogen	A‐11029	Goat	Alexa Fluor 488	Immunocytochemistry	1:1,000
Mouse IgG	Invitrogen	A‐32723	Goat	Alexa Fluor Plus 488	Immunocytochemistry	1:1,000
Mouse IgG	Invitrogen	A‐10037	Donkey	Alexa Fluor 568	Immunocytochemistry	1:1,000
					Immunohistochemistry	1:1,000
Mouse IgG	Invitrogen	A‐32728	Goat	Alexa Fluor Plus 647	Immunocytochemistry	1:1,000
Mouse IgG_1_	Invitrogen	A‐21121	Goat	Alexa Fluor 488	Immunocytochemistry	1:1,000
Mouse IgG_1_	Invitrogen	A‐21125	Goat	Alexa Fluor 594	Immunocytochemistry	1:1,000
					Immunohistochemistry	1:1,000
Mouse IgG_1_	Invitrogen	A‐21240	Goat	Alexa Fluor 647	Immunocytochemistry	1:1,000
					Immunohistochemistry	1:1,000
Mouse IgG_2a_	Invitrogen	A‐21131	Goat	Alexa Fluor 488	Immunocytochemistry	1:1,000
Mouse IgG_2a_	Invitrogen	A‐21135	Goat	Alexa Fluor 594	Immunocytochemistry	1:1,000
Mouse IgG_2a_	Invitrogen	A‐21241	Goat	Alexa Fluor 647	Immunocytochemistry	1:1,000
Rabbit IgG	Vector Laboratories	BA‐1000	Goat	Biotin	Immunohistochemistry	1:1,000
Rabbit IgG	Invitrogen	A‐11008	Goat	Alexa Fluor 488	Immunohistochemistry	1:1,000
Rabbit IgG	Invitrogen	A‐11034	Goat	Alexa Fluor 488	Immunocytochemistry	1:1,000
Rabbit IgG	Invitrogen	A‐32731	Goat	Alexa Fluor Plus 488	Immunocytochemistry	1:1,000
					Immunohistochemistry	1:1,000
Rabbit IgG	Invitrogen	A‐10042	Donkey	Alexa Fluor 568	Immunocytochemistry	1:1,000
Rabbit IgG	Invitrogen	A‐11037	Goat	Alexa Fluor 594	Immunocytochemistry	1:1,000
Rabbit IgG	Invitrogen	A‐32733	Goat	Alexa Fluor Plus 647	Immunocytochemistry	1:1,000
Rat IgG	Invitrogen	A‐11006	Goat	Alexa Fluor 488	Immunocytochemistry	1:1,000
Rat IgG	Invitrogen	A‐11007	Goat	Alexa Fluor 594	Immunocytochemistry	1:1,000

### 5.10 Immunocytochemistry

Immunocytochemistry of cultured cells was performed as previously described [31]. Briefly, cells were fixed with 4% paraformaldehyde (PFA; FUJIFILM Wako Pure Chemical, 163–20145) for 10 min at room temperature, washed with PBS, and permeabilized and blocked for 15–20 min at room temperature with PBS containing Ca^2+^/Mg^2+^ (Corning, 21‐030‐CV), 0.5% (w/v) BSA (GenDEPOT, A0100‐005), and 0.1% Triton X‐100 (Sigma–Aldrich, T8787) (hereafter referred to as the blocking solution I). Primary antibodies diluted in the blocking solution I were applied overnight at 4°C. After three washes with PBS, cells were incubated with Alexa Fluor 488‐, 568‐, 594‐, or 647‐conjugated secondary antibodies in blocking solution I for 2 h at room temperature. All antibodies used, including dilution details, are listed in Table 2. Nuclei were counterstained with 1 µg/mL 4′,6‐diamidino‐2‐phenylindole (DAPI; Invitrogen, D1306) for 7–10 min. After three additional washes with PBS, cells were imaged either directly in PBS or after mounting with mounting medium (Vector Laboratories, H‐1400‐10) and coverslips. Images were acquired using fluorescence microscopy (Lionheart FX; BioTek) or confocal microscopy (STELLARIS 5 LIA, Leica; LSM 900 with Airyscan 2, Carl Zeiss).

For cryosection‐based immunostaining of neurospheroids (Figure S8H), samples were fixed in 4% PFA for 15 min at room temperature, washed with PBS, cryoprotected in 30% (w/v) sucrose in PBS, embedded in OCT compound (Sakura Finetek, 4583), cryosectioned at 10 µm thickness, and mounted on glass slides. Sections were permeabilized and blocked in blocking solution I for 15–20 min at room temperature, incubated overnight at 4°C with primary antibodies in blocking solution I, washed three times with PBS, and incubated with Alexa Fluor‐conjugated secondary antibodies in blocking solution I for 2 h at room temperature. Nuclei were counterstained with 1 µg/mL DAPI for 7–10 min, washed three times with PBS, and imaged after mounting with mounting medium and coverslips, using fluorescence or confocal microscopy.

For whole‐mount immunostaining of neurospheroids (Figure 5J), samples were rendered optically transparent using the Binaree Tissue Clearing Kit (Binaree Inc., HRTC‐012) according to the manufacturer's optimized protocol. Following tissue clearing, neurospheroids were subjected to immunostaining with primary and Alexa Fluor‐conjugated secondary antibodies as described above. After nuclear counterstaining with DAPI, cleared neurospheroids were imaged using confocal microscopy.

### 5.11 Immunohistochemistry

Mice were deeply anesthetized and transcardially perfused with PBS, followed by 4% PFA in PBS. Brains were carefully isolated and post‐fixed in 4% PFA at 4 °C for 16 h. After fixation, tissues were cryoprotected by immersion in 30% (w/v) sucrose in PBS for 3 days at 4 °C until they sank. The brains were then embedded and rapidly frozen on dry ice. Coronal sections were cut at a thickness of 35 µm using a freezing microtome (Thermo Fisher Scientific, HM430).

For 3,3’‐diaminobenzidine (DAB) staining, floating sections were blocked with blocking solution II [5% normal goat serum (Invitrogen, 16201), 0.1% Triton X‐100 (Sigma–Aldrich, X100) in PBS, pH 7.4] for 1 h at room temperature. The tissues were incubated with rabbit anti‐TH primary antibody (1:5,000; Novus Biologicals, NB300‐109) at 4°C for 16 h. The next day, the samples were washed three times with wash solution [0.1% Triton X‐100 in PBS, pH 7.4] and incubated with a biotinylated secondary antibody (1:1000; Vector Laboratories, BA‐1000). Sections were then incubated with ABC reagent and developed with DAB (Sigma–Aldrich, D4293) as the HRP substrate. Immunostained brain sections were counterstained with Nissl. The total number of TH‐positive neurons in the SNpc was quantified using the optical fractionator stereological probe in Stereo Investigator software (MBF Bioscience). All stereological analyses were performed by an investigator blinded to the experimental treatment groups.

For immunofluorescence staining, mouse coronal sections fixed in 4% PFA were blocked with blocking solution II for 1 h at room temperature. Sections were then incubated with primary antibodies at 4°C overnight. The following day, floating brain sections were washed three times with wash solution and incubated with corresponding secondary antibodies conjugated to fluorescent dyes at room temperature for 1 h. All antibodies used, including dilution details, are listed in Table 2. After washing three times with wash solution, sections were incubated with DAPI (Sigma–Aldrich, D9542) solution for nuclear staining. Sections were then washed with PBS and mounted onto microscope slides using mounting medium (Thermo Fisher Scientific, 9990402). Fluorescence images were acquired using either an Axio Imager M2 fluorescence microscope (Carl Zeiss) equipped with appropriate filter sets or a STELLARIS 5 LIA confocal microscope (Leica).

### 5.12 Patch‐Clamp Electrophysiology

hESC‐derived vmDA neurons were recorded in artificial cerebrospinal fluid (aCSF) continuously perfused at a flow rate of 2 mL/min. The aCSF consisted of 130 mm NaCl, 24 mm NaHCO_3_, 3.5 mm KCl, 1.25 mm NaH_2_PO_4_, 1.5 mm MgCl_2_, 1.5 mm CaCl_2_, and 10 mm d‐(+)‐glucose, adjusted to pH 7.4. The recording chamber was mounted on the stage of an inverted microscope (IX51; Olympus). Cell morphology was visualized using a charge‐coupled device camera (iXon Life 897 EMCCD; Andor Technology), and images were captured and processed using Imaging Workbench software (Indec BioSystems). Patch electrodes (6–8 MΩ) were filled with an internal solution containing 150 mm KCl, 1 mm CaCl_2_, 1 mm MgCl_2_, 5 mm EGTA, and 10 mm HEPES (pH adjusted to 7.4 with KOH). Cells were subjected to a series of tests of gap‐free recording for spontaneous currents, current‐step tests, and voltage‐step tests. Gap‐free currents were recorded under voltage clamp at a holding potential (V_h) of −60 mV. Current‐step tests were conducted by delivering 1‐s current pulses from −40 pA to +250 pA in 10‐pA increments. Voltage‐step tests were performed by holding cells at −60 mV and applying 1‐s voltage steps to test potentials ranging from −80 to +100 mV in 10‐mV increments. Electrical signals were amplified and low‐pass filtered at 2 kHz using a MultiClamp 700B amplifier (Molecular Devices) and digitized at 10 kHz using a Digidata 1440A interface (Molecular Devices). Cell membrane capacitance was measured using the ‘membrane test’ protocol built into pClampex software (Molecular Devices).

### 5.13 Dopamine (DA) ELISA

The concentration of DA in the culture supernatants was measured as previously described [31]. Cultured hESC‐derived vmDA neurons and cortical neurons were washed with HBSS (Gibco, 14025092), then incubated in fresh HBSS for 15 min, after which supernatants were collected as *controls*. Subsequently, cells were incubated in HBSS with or without 50 mm KCl (Sigma–Aldrich, 60142‐100ML‐F) for an additional 15 min, and these supernatants were collected as *samples*. Immediately following collection, 1 mm EDTA (Invitrogen, 15575020) and 4 mm sodium metabisulfite (Sigma–Aldrich, S9000) were added to the samples to prevent DA degradation. Samples were stored at −80°C until analysis. DA concentrations were quantified using the DOPAMINE high‐sensitivity ELISA (LDN, BA E‐5300R) according to the manufacturer's instructions. DA release levels were calculated by subtracting DA concentrations in the *controls* from those in the corresponding *samples*.

### 5.14 Animal Experiments

All animal experiments were approved by the ethical committee of Sungkyunkwan University (IACUC 2024‐10‐32‐1) and were conducted in accordance with all applicable international guidelines. DAT‐PF‐tTA mice were purchased from Jackson Laboratory (strain #027178). A plasmid encoding β23 under the control of the tetracycline‐responsive promoter (TetP) was constructed and purified, then submitted to Macrogen Inc. for generation of transgenic (Tg) mice by pronuclear microinjection. Genotyping was performed by PCR using genomic DNA extracted from tail biopsies of pups (DirectPCR Lysis Reagent; Viagen Biotech, 102‐T). Primer sequences used for genotyping are listed in Table 1. Mice were backcrossed to a C57BL/6 background, and both male and female littermates were used in all experiments, and all animals included in the study were analyzed. The number of mice used for each experiment was determined based on previous studies in the same research field using similar technical approaches. No randomization was performed for colony maintenance or group allocation. Experimenters conducting behavioral and neuropathological analyses were blinded to the experimental group of each mouse. To suppress transgene expression during development, mice were maintained on a doxycycline‐containing diet (200 mg/kg diet; DooYeol Biotech, DY‐DM‐5700) ad libitum until 3 weeks of age. Transgene expression was then induced by switching mice to regular chow and maintained until the indicated analysis.

### 5.15 Stereotaxic Cell Transplantation

Stereotaxic transplantation of hESC‐derived vmDA progenitors was performed in DAT‐PF‐tTA;TetP‐β23 (β23 Tg) mice at 14 weeks of age, following transgene induction initiated at 3 weeks of age. After sterilization of the incision area with 70% ethanol, a small (∼0.5 cm) incision was made in the scalp. A small burr hole was drilled in the skull to target the striatum, and a 25‐gauge cannula was bilaterally positioned into the striatum using the following stereotaxic coordinates relative to bregma: anteroposterior, +0.5 mm; mediolateral, ±1.5 mm; dorsoventral, −2.5 mm. A total of 1.5 µL of vmDA cell suspension (1.5 × 10^5^ cells) was infused per site at a rate of 0.2 µL/min. Bilateral injections resulted in a total of 3.0 × 10^5^ cells delivered per mouse. To aid diffusion and minimize backflow, the injection cannula was left in place for 10 min after the final infusion, during which the syringe was incrementally retracted to reduce cell reflux before slowing complete withdrawal. Surgical wounds were closed with sutures, and the animals were monitored daily for wound healing and recovery throughout the experimental period. Immunosuppression was administered using cyclosporine A at a dose of 10 mg/kg via intraperitoneal injection once daily, starting three days prior to transplantation and continuing until the time of sacrifice.

### 5.16 Behavior Tests

(Rotarod test) Mice were habituated to the rotarod apparatus for one day prior to testing. On the test day, mice were placed on the rotating cylinder, and the latency to fall and the speed at which they fell were recorded. The rotarod speed continuously increased from 4 to 40 revolutions per minute (rpm) over a 5‐min period. The trial was terminated when the mouse fell from the rod or reached the maximum test duration of 5 min, whichever occurred first. (Pole test) The pole consisted of a vertical metal rod (58 cm long, 10 mm diameter) wrapped with tape to provide grip. For the pole test, mice were first acclimated to the test cage for at least 5 min. Once acclimated, mice were gently placed at the top of the pole facing downward. The time required for the mouse to descend and place its forelimbs on the floor was recorded as the latency to descend. Three consecutive trials were performed for each mouse with 5‐min intervals between trials, and the average latency was calculated.

### 5.17 Statistical Analysis

Data were analyzed using Prism 10.5 software (GraphPad) and are presented as mean ± standard deviation (SD). Technical replicates, when performed, were averaged within each biological replicate before statistical analysis and were not treated as independent observations. The sample size (*n*) and experimental unit for each analysis are specified in the corresponding figure legends. Statistical significance between two groups was assessed using a two‐tailed unpaired Student's *t*‐test. Comparisons among more than two groups were analyzed by one‐way or two‐way ANOVA followed by Tukey's or Šidák's multiple comparisons tests, as indicated in the figure legends. Differences were considered statistically significant at *p* < 0.05.

## Author Contributions

Conceptualization, data curation, visualization, writing – original draft: H.M., He.K., E.H.L., Ha.K., J.‐W.C., S.‐J.O., Y.L., and Y.O. Investigation, methodology: H.M., He.K., E.H.L., Ha.K., J.H.K., H.J., J.C., G.R.J., B.D.L., and J.‐W.C. Project administration, resources: S.‐J.O., Y.L., and Y.O. Funding acquisition: Y.L. and Y.O. Supervision: Y.O.

## Funding

This work was supported by the Korea Fund for Regenerative Medicine funded by the Ministry of Science and ICT (MSIT) and Ministry of Health and Welfare (RS‐2026‐25502371, Y.O.), the National Research Foundation of Korea (NRF) grants funded by MSIT (RS‐2020‐NR048009, Y.O.; RS‐2024‐00349023, Y.L.) and the K‐Brain Project of the NRF funded by MSIT (RS‐2023‐00266171, Y.O.). Additional support was provided by the Korea Basic Science Institute (National Research Facilities and Equipment Center) grant funded by the Ministry of Education (RS‐2023‐NF001313, Y.O.).

## Conflicts of Interest

H.M., He.K., E.H.L., Ha.K., S.‐J.O., Y.L., and Y.O. are named inventors on patent applications related to this work filed by Hanyang University (Korean patent application no. 10‐2026‐0126364, pending; PCT application no. PCT/KR2026/008964, pending). The authors declare no conflicts of interest other than the patent applications disclosed above.

## Supporting information




**Supporting File 1**: advs78007‐sup‐0001‐SuppMat.pdf.

**Supporting File 2**: advs78007‐sup‐0002‐xlsx.zip.

## Data Availability

The raw RNA‐seq data generated in this study have been deposited in the Gene Expression Omnibus (GEO) under accession number GSE319963. Processed RNA‐seq data supporting the findings of this study are provided in Tables S1–S6. The scripts used for bulk RNA‐seq data processing and downstream analyses are available at Zenodo (https://doi.org/10.5281/zenodo.18690462). Additional data supporting the conclusions of this study are available from the corresponding author upon reasonable request.
