# Supplementary material for: Developmentally Guided Differentiation of Ventral Midbrain Dopaminergic Cells Validated in Proteopathy‐Based Parkinson's Disease Models
Source: Adv Sci (Weinh). 2026 Sep 29:e78007. Online ahead of print. doi: 10.1002/advs.78007 (PMC13624424; doi:10.1002/advs.78007)
Supplement: Supplementary file 1 [file ADVS-9999-e78007-s002.pdf]

Developmentally guided differentiation of ventral midbrain dopaminergic cells validated in proteopathy-based Parkinson's disease models

Heechang Moon[†], Heejeong Kim[†,‡], Elliot H. Lee, Hanseul Kim, Ji Hun Kim, Huisu Jeong, Jiwon Cheon, Ga Ram Jeong, Byoung Dae Lee, Jae-Won Cho, Soo-Jin Oh, Yunjong Lee[*], and Yohan Oh[*]

H. Moon, Ha. Kim, H. Jeong, Y. Oh
Department of Biomedical Science, Graduate School of Biomedical Science and Engineering, Hanyang University, Seoul 04763, Republic of Korea
E-mail: yoh@hanyang.ac.kr

He. Kim, J. H. Kim, J. Cheon, Y. Lee
Department of Pharmacology, Samsung Biomedical Research Institute, Sungkyunkwan University School of Medicine, Suwon 16419, Republic of Korea
E-mail: ylee69@skku.edu

E. H. Lee, S.-J. Oh
Brain Science Institute, Korea Institute of Science and Technology, Seoul 02792, Republic of Korea

E. H. Lee
Department of Biotechnology, College of Life Science and Biotechnology, Yonsei University, Seoul 03722, Republic of Korea

Ha. Kim, J.-W. Cho, Y. Oh
Hanyang Institute of Bioscience and Biotechnology, Hanyang University, Seoul 04763, Republic of Korea

G. R. Jeong, B. D. Lee
Department of Neuroscience, Kyung Hee University, Seoul 02447, Republic of Korea

B. D. Lee
Department of Physiology, Kyung Hee University, Seoul 02447, Republic of Korea

J.-W. Cho
Department of Life Science, College of Natural Sciences, Hanyang University, Seoul 04763, Republic of Korea

Y. Oh
Department of Biochemistry and Molecular Biology, College of Medicine, Hanyang University, Seoul 04763, Republic of Korea

Y. Oh
Hanyang Institute of Advanced BioConvergence, Hanyang University, Seoul 04763, Republic of Korea

Funding: This work was supported by the Korea Fund for Regenerative Medicine funded by the Ministry of Science and ICT (MSIT) and Ministry of Health and Welfare (RS-2026-25502371, Y.O.), the National Research Foundation of Korea (NRF) grants funded by MSIT (RS-2020-NR048009, Y.O.; RS-2024-00349023, Y.L.) and the K-Brain Project of the NRF funded by MSIT (RS-2023-00266171, Y.O.). Additional support was provided by the Korea Basic Science Institute (National research Facilities and Equipment Center) grant funded by the Ministry of Education (RS-2023-NF001313, Y.O.).

Keywords: Differentiation protocol; ventral midbrain dopaminergic progenitor; ventral midbrain dopaminergic neuron; human pluripotent stem cell; Parkinson's disease; cell therapy

Footnotes

† Heechang Moon and Heejeong Kim contributed equally to this work.

‡ Present address of Heejeong Kim: Bio Healthcare Central Research Center, Hyper Corporation Inc., Seoul 06047, Republic of Korea

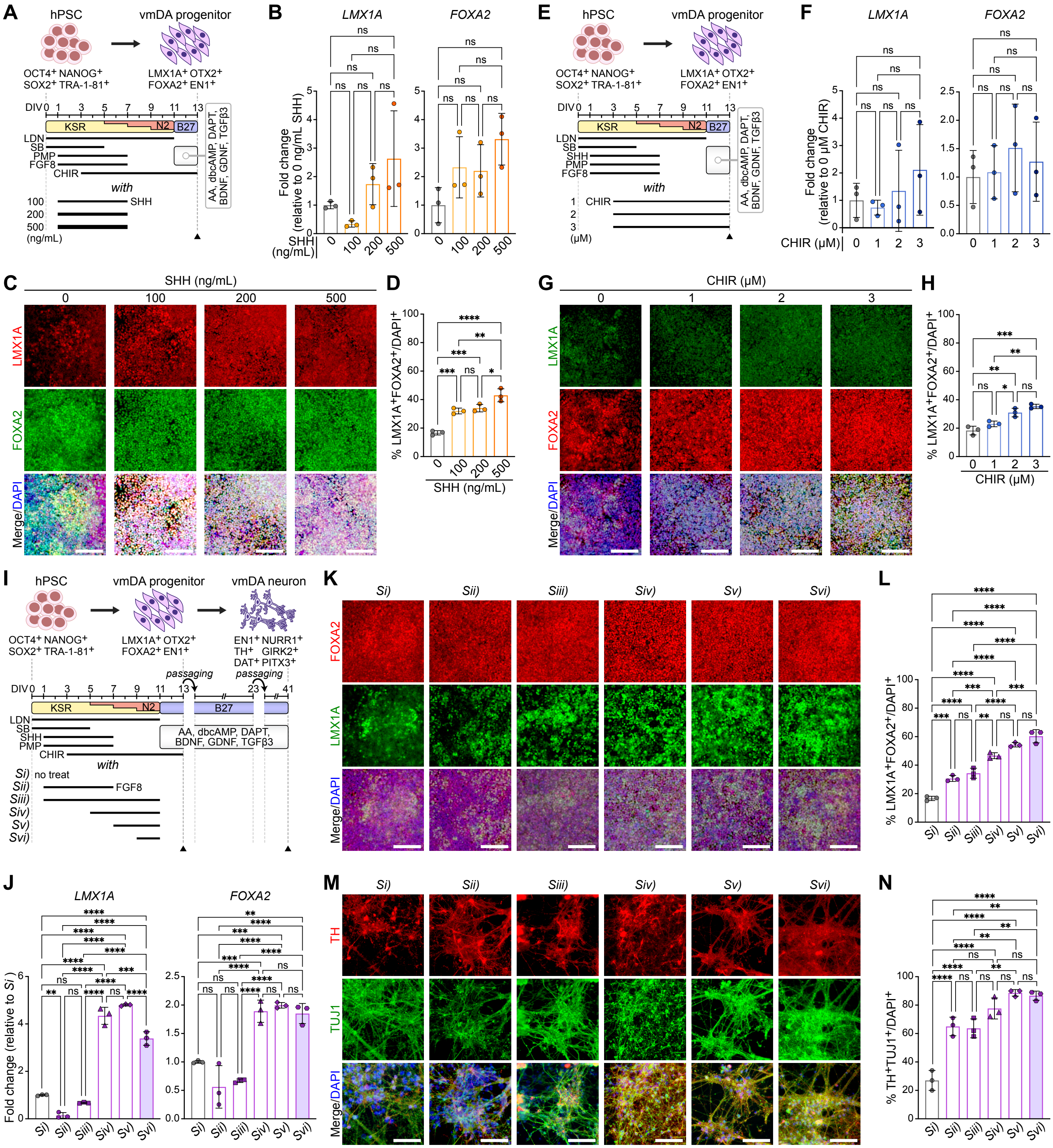

Figure S1. Effects of SHH, WNT, and FGF8 signaling parameters on vmDA progenitor specification and dopaminergic differentiation. (**A**) Schematic overview of the culture conditions and differentiation timeline used for vmDA progenitor induction and subsequent neuronal maturation. (**B**) RT–qPCR analysis of *LMX1A* and *FOXA2* expression in DIV 13 progenitors following SHH titration (0–500 ng/mL) (*n* = 3 biological replicates). Data are shown as fold change relative to 0 ng/mL SHH. (**C**) Representative immunofluorescence images of DIV 13 progenitors stained for LMX1A and FOXA2 under the indicated SHH concentrations. Nuclei were counterstained with DAPI. (**D**) Quantification of LMX1A/FOXA2 double-positive progenitors among total DAPI^{+} cells following SHH titration (*n* = 3 biological replicates). (**E**) Schematic illustrating the experimental design for CHIR99021 (CHIR) titration during vmDA progenitor differentiation. (**F**) RT–qPCR analysis of

LMX1A and *FOXA2* expression in DIV 13 progenitors following CHIR titration (0–3 µM) ($n = 3$ biological replicates). Data are shown as fold change relative to 0 µM CHIR. (**G**) Representative immunofluorescence images of DIV 13 progenitors stained for LMX1A and FOXA2 under the indicated CHIR concentrations. (**H**) Quantification of LMX1A/FOXA2 double-positive progenitors among total $DAPI^+$ cells following CHIR titration ($n = 3$ biological replicates). (**I**) Experimental timelines illustrating differentiation protocols (*Si*–*Svi*) used to assess the effects of altering the onset and duration of FGF8 treatment during vmDA progenitor specification. (**J**) RT–qPCR analysis of *LMX1A* and *FOXA2* expression in DIV 13 progenitors generated under the indicated FGF8 timing conditions; data are shown as fold change relative to *Si* ($n = 3$ biological replicates). (**K**) Representative immunofluorescence images of DIV 13 progenitors stained for LMX1A and FOXA2 under the indicated FGF8 timing conditions. (**L**) Quantification of LMX1A/FOXA2 double-positive progenitors among total $DAPI^+$ cells under each FGF8 timing condition ($n = 3$ biological replicates). (**M**) Representative immunofluorescence images of differentiated neurons at DIV 41 stained for TH and TUJ1 following vmDA progenitor generation under the indicated FGF8 timing conditions. (**N**) Quantification of TH/TUJ1 double-positive neurons among total $DAPI^+$ cells at DIV 41 ($n = 3$ biological replicates). Arrowheads indicate the time points for downstream experiments. Scale bars, 100 µm. Data are presented as mean ± SD. Statistical significance was determined by one-way ANOVA with Tukey's multiple-comparison test. ns, not significant; $^*p < 0.05$, $^{**}p < 0.01$, $^{***}p < 0.001$, $^{****}p < 0.0001$.

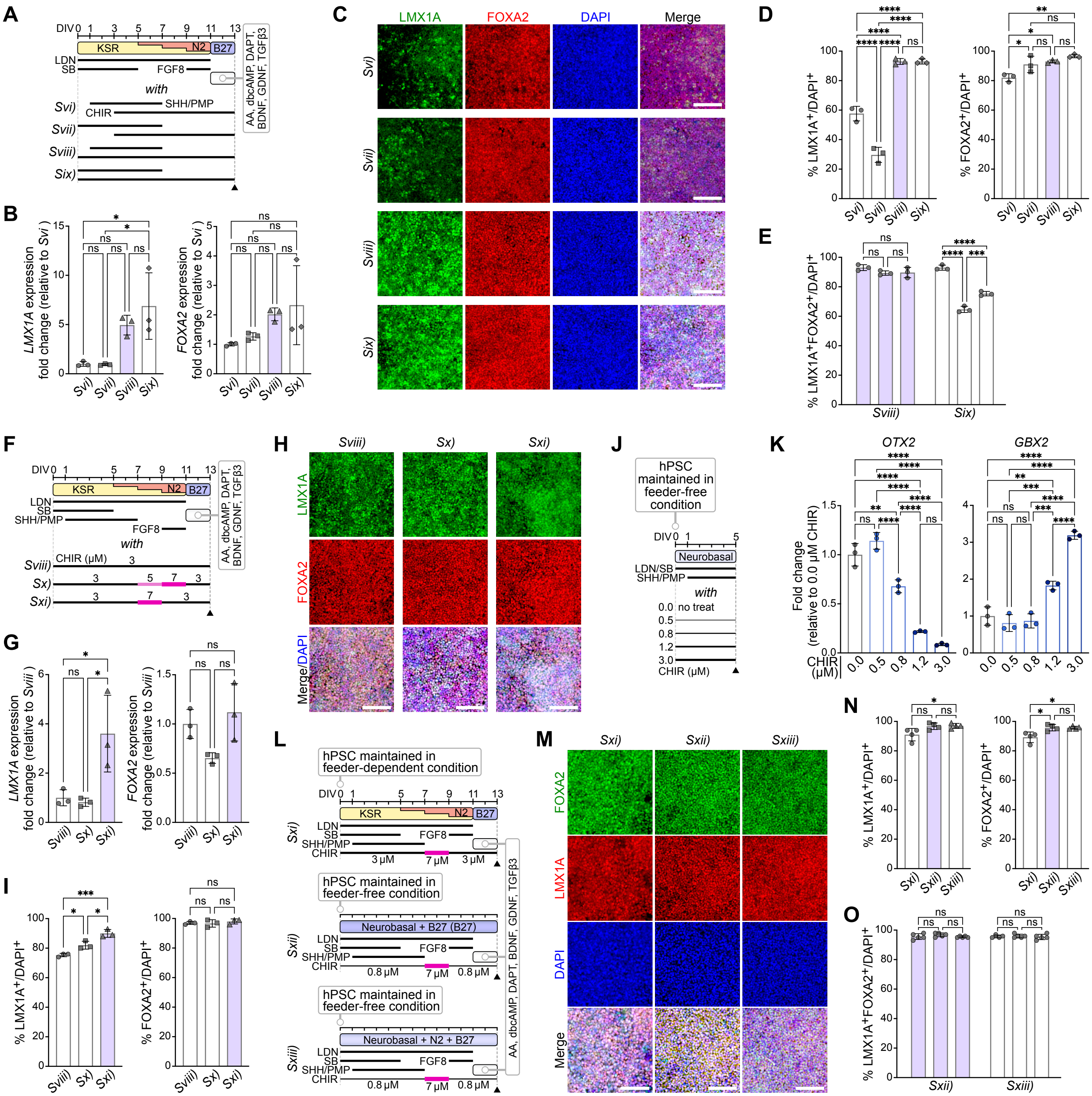

Figure S2. Effects of SHH and WNT signaling onset timing and culture conditions on vmDA progenitor specification. (**A**) Schematic comparison of differentiation protocols differing in the onset of SHH/PMP and CHIR treatment. (**B**) RT–qPCR analysis of *LMX1A* and *FOXA2* expression in DIV 13 progenitors; data are shown as fold change relative to *Svi* ($n = 3$ biological replicates). (**C**) Representative immunofluorescence images of DIV 13 progenitors stained for LMX1A and FOXA2; nuclei were counterstained with DAPI. (**D**) Quantification of LMX1A$^+$ and FOXA2$^+$ progenitors among total DAPI$^+$ cells ($n =$ 3 biological replicates). (**E**) Batch-to-batch comparison of LMX1A/FOXA2 double-positive progenitor yield generated using *Sviii* and *Six* protocols across three independent differentiation batches. Each bar corresponds to one differentiation batch, and dots represent three independently established cultures (biological replicates) within that batch. ($n = 3$ biological replicates per batch). (**F**) Schematic illustrating CHIR boosting conditions. (**G**) RT–qPCR analysis of *LMX1A* and *FOXA2* expression in DIV 13 progenitors; data are shown as fold change relative to *Sviii* ($n = 3$ biological replicates). (**H**) Representative immunofluorescence images of DIV 13 progenitors stained for LMX1A and FOXA2. (**I**) Quantification of LMX1A$^+$ and FOXA2$^+$ progenitors among total DAPI$^+$ cells ($n = 3$ biological replicates). (**J**) Schematic of CHIR titration under KSR-free

conditions to assess regional identity. (**K**) RT–qPCR analysis of *OTX2* and *GBX2* expression in DIV 5 cells (n = 3 biological replicates). (**L**) Schematic illustrating the comparison between feeder-dependent (KSR-based) and feeder-free (Neurobasal-based) culture conditions during vmDA progenitor specification. (**M**) Representative immunofluorescence images of DIV 13 progenitors stained for LMX1A and FOXA2. (**N**) Quantification of $LMX1A^+$ and $FOXA2^+$ progenitors among total $DAPI^+$ cells (n = 4 biological replicates). (**O**) Batch-to-batch comparison of LMX1A/FOXA2 double-positive progenitor yield across three independent differentiation batches under each indicated condition. Each bar corresponds to one differentiation batch, and dots represent five independently established cultures (biological replicates) within that batch. (n = 5 biological replicates per batch). Arrowheads indicate the time points for downstream experiments. Scale bars, 100 μm. Data are presented as mean ± SD. Statistical significance was determined by one-way ANOVA with Tukey's multiple-comparison test. ns, not significant; $*p < 0.05$, $**p < 0.01$, $***p < 0.001$, $****p < 0.0001$.

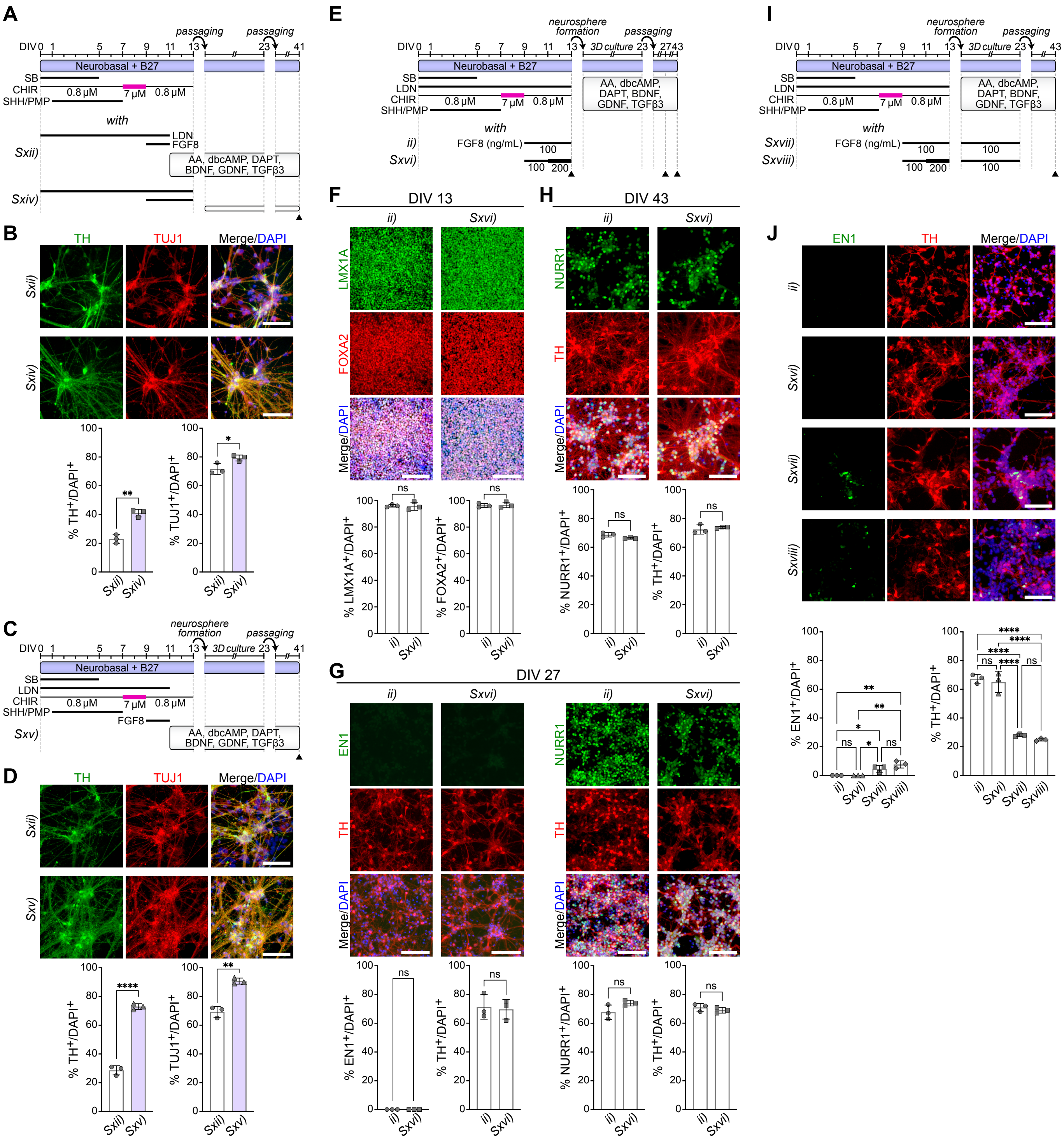

Figure S3. FGF8 modulation and three-dimensional maturation resulted in barely detectable EN1 induction under baseline patterning conditions. (**A**) Schematic illustration of differentiation conditions used to examine the effects of FGF8 exposure under baseline vmDA patterning. (**B**) Representative immunofluorescence images of neurons at DIV 41 stained for TH and TUJ1, with quantification of TH^+ and $TUJ1^+$ cells among total $DAPI^+$ cells (n = 3 biological replicates). (**C**) Schematic overview of three-dimensional (3D) neurosphere-based differentiation protocols. (**D**) Representative immunofluorescence images of neurons at DIV 41 stained for TH and TUJ1, with quantification of TH^+ and $TUJ1^+$ cells among total $DAPI^+$ cells (n = 3 biological replicates). (**E**) Schematic illustration of differentiation protocols incorporating biphasic FGF8 treatment under baseline vmDA patterning conditions. (**F**) Representative immunofluorescence images of progenitors at DIV 13 stained for LMX1A and FOXA2, with quantification of $LMX1A^+$ and $FOXA2^+$ cells among total $DAPI^+$ cells (n = 3 biological replicates).

(**G**) Representative immunofluorescence images of neurons at DIV 27 stained for EN1 and TH or for NURR1 and TH, with quantification of $EN1^+$, $NURR1^+$, and TH^+ cells among total $DAPI^+$ cells (n = 3 biological replicates). (**H**) Representative immunofluorescence images of neurons at DIV 43 stained for NURR1 and TH, with quantification of $NURR1^+$ and TH^+ cells among total $DAPI^+$ cells (n = 3 biological replicates). (**I**) Schematic illustrating extended FGF8 exposure under baseline vmDA patterning conditions. (**J**) Representative immunofluorescence images of neurons at DIV 43 stained for EN1 and TH, with quantification of $EN1^+$ and TH^+ cells among total $DAPI^+$ cells (n = 3 biological replicates). Arrowheads indicate the time points for downstream experiments. Scale bars, 100 µm. Data are presented as mean ± SD. Statistical significance was determined by two-tailed unpaired Student's t-test (**B,D,F–H**) or one-way ANOVA with Tukey's multiple-comparison test (**J**). ns, not significant; $*p < 0.05$, $**p < 0.01$, $****p < 0.0001$.

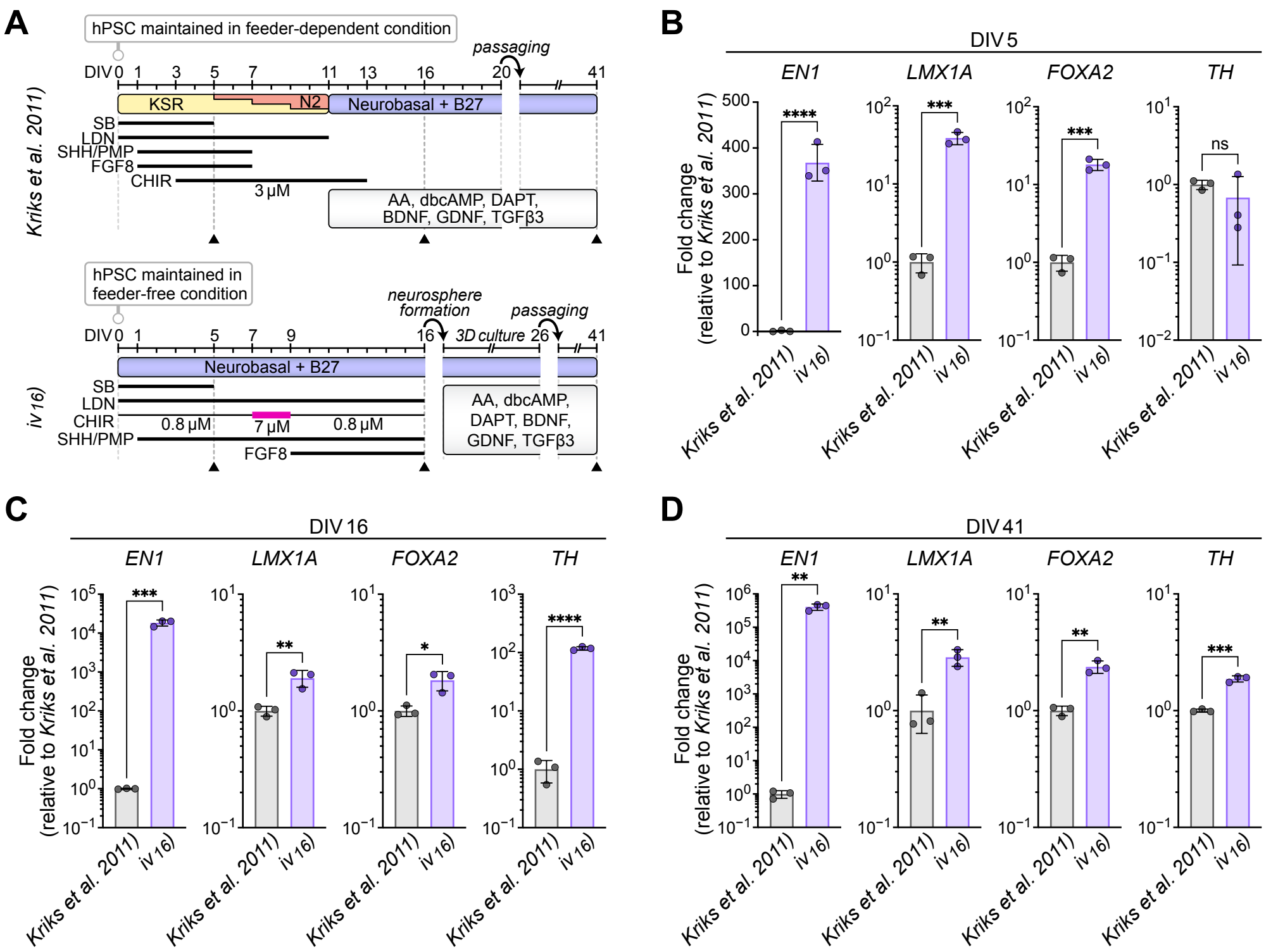

Figure S4. Comparison of vmDA marker expression between the Kriks et al. 2011 protocol and a modified vmDA differentiation protocol. (**A**) Schematic illustration of a reference vmDA differentiation protocol (Kriks et al., 2011) and the modified protocol incorporating the indicated changes. (**B–D**) RT-qPCR analysis of vmDA lineage marker expression (*EN1*, *LMX1A*, *FOXA2*, and TH*)* under the reference and modified differentiation conditions at DIV 5 (**B**), at DIV 16 corresponding to the end of the patterning phase (**C**), and at DIV 41 following neuronal differentiation (**D**) ($n = 3$ biological replicates). Arrowheads indicate the time points for downstream experiments. Data are presented as mean ± SD. Statistical significance was determined by two-tailed unpaired Student's *t*-test. ns, not significant; *$p < 0.05$, **$p < 0.01$, ***$p < 0.001$, ****$p < 0.0001$.

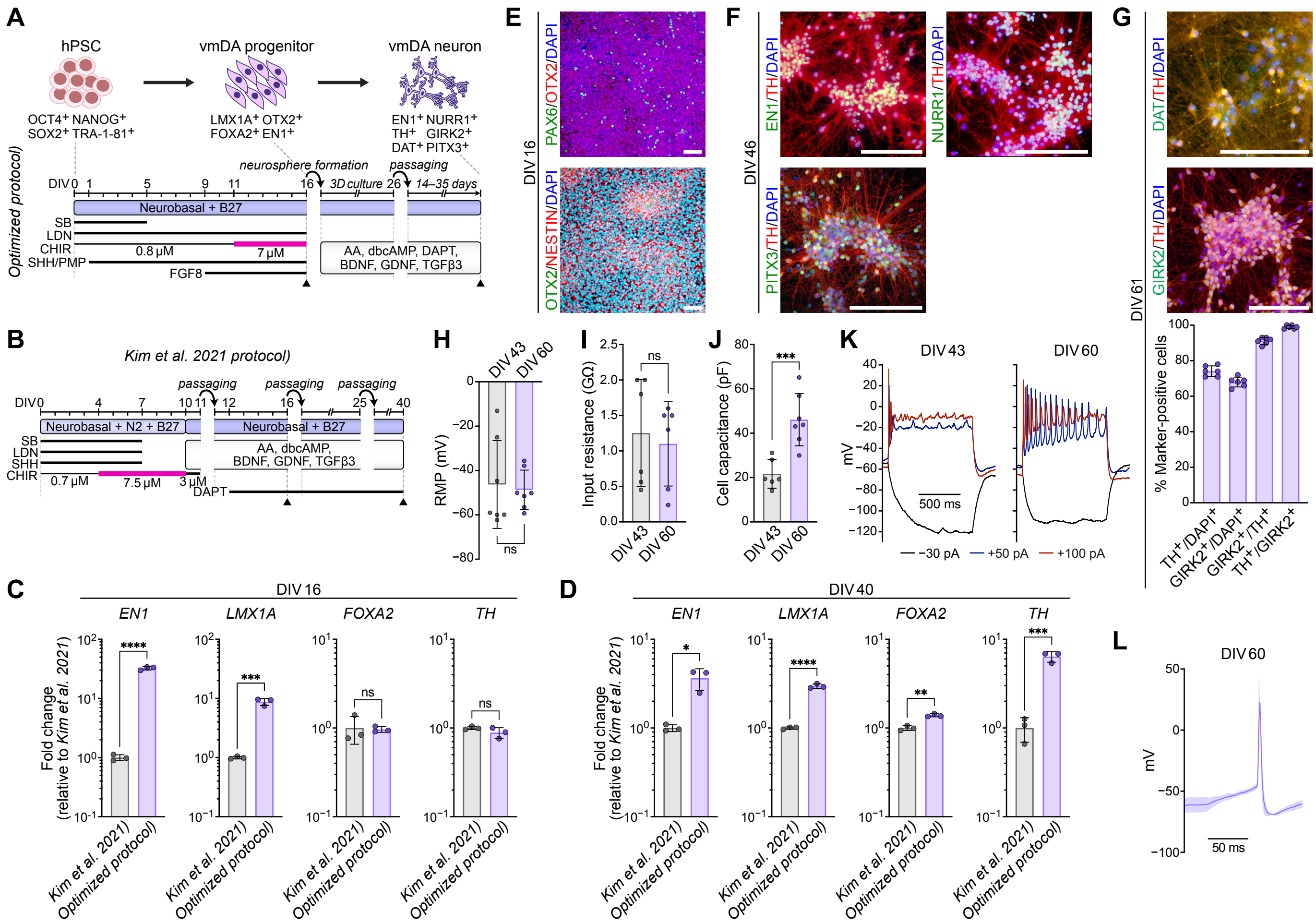

Figure S5. Comparison of vmDA marker expression between the Kim et al. 2021 protocol and the optimized protocol, and molecular and electrophysiological characterization of vmDA lineage cells generated using the optimized protocol. (**A**) Schematic overview of the optimized vmDA differentiation protocol. (**B**) Schematic overview of the Kim et al. 2021 protocol used for direct comparison. (**C,D**) RT-qPCR analysis of vmDA lineage marker expression under the Kim et al. 2021 protocol and the optimized protocol at DIV 16 corresponding to the vmDA progenitor stage (**C**) and DIV 40 following neuronal differentiation (**D**) ($n = 3$ biological replicates). (**E**) Representative immunofluorescence images at DIV 16 stained for PAX6, OTX2, and NESTIN. (**F**) Representative immunofluorescence images at DIV 46 showing expression of EN1, NURR1, and PITX3 together with TH. (**G**) Representative immunofluorescence images at DIV 61 showing expression of DAT and GIRK2 together with TH. Quantification shows the percentages of TH^+ and $GIRK2^+$ cells among total $DAPI^+$ cells, $GIRK2^+$ cells among TH^+ cells, and TH^+ cells among $GIRK2^+$ cells ($n = 6$ biological replicates). (**H–J**) Passive electrophysiological properties of vmDA neurons recorded at DIV 43 and DIV 60, including resting membrane potential (RMP; **H**), input resistance (**I**), and cell capacitance (**J**). All recordings were obtained from three biological replicates. Sample sizes were $n = 7$ neurons for **H**, $n = 6$ neurons for **I**, and $n = 6$ neurons (DIV 43) and $n = 7$ neurons (DIV 60) for **J**. (**K**) Representative voltage responses to step current injections at DIV 43 and DIV 60. (**L**) Average traces of single action potentials evoked at rheobase current injection recorded at DIV 60 ($n = 4$ neurons from 3 biological replicates). Arrowheads indicate the time points for downstream experiments. Scale bars, 100 µm. Data are presented as mean ± SD. Statistical significance was determined by two-tailed unpaired Student's *t*-test. ns, not significant; $*p < 0.05$, $**p < 0.01$, $***p < 0.001$, $****p < 0.0001$.

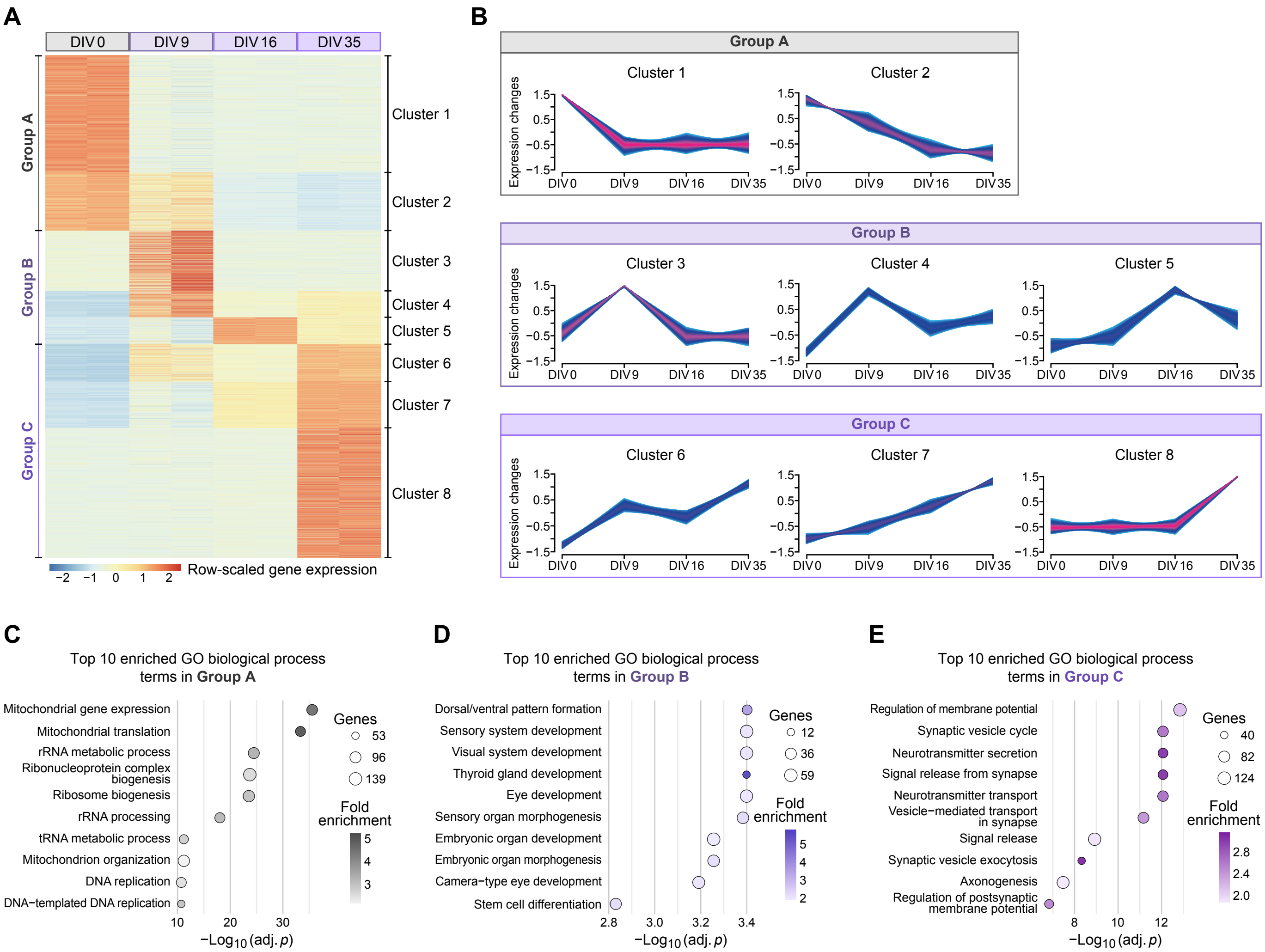

Figure S6. Temporal gene expression dynamics and hierarchical organization of transcriptional programs during vmDA differentiation. (**A**) Row-scaled heatmap of protein-coding genes clustered by Mfuzz across DIV 0, DIV 9, DIV 16, and DIV 35 ($n = 2$ biological replicates per time point), with assignment of eight temporal clusters into three higher-order transcriptional programs (Groups A–C). (**B**) Temporal expression trajectories of the eight Mfuzz clusters grouped according to shared dynamics into Group A (early downregulated), Group B (transient/intermediate), and Group C (late upregulated). (**C**) Top 10 enriched Gene Ontology (GO) Biological Process terms in Group A, representing a pluripotency-associated biosynthetic and proliferative program that is progressively downregulated during differentiation. (**D**) Top 10 enriched GO Biological Process terms in Group B, representing a developmental patterning and morphogenetic program; this group includes *EN1* (cluster 5), consistent with acquisition of ventral midbrain competence. (**E**) Top 10 enriched GO Biological Process terms in Group C, representing a neuronal functional maturation program enriched for synaptic transmission, membrane excitability, regulated exocytosis, and dopaminergic synapse–associated pathways. Complete enrichment results for GO, KEGG, Reactome, and MSigDB analyses are provided in Table S1–S4.

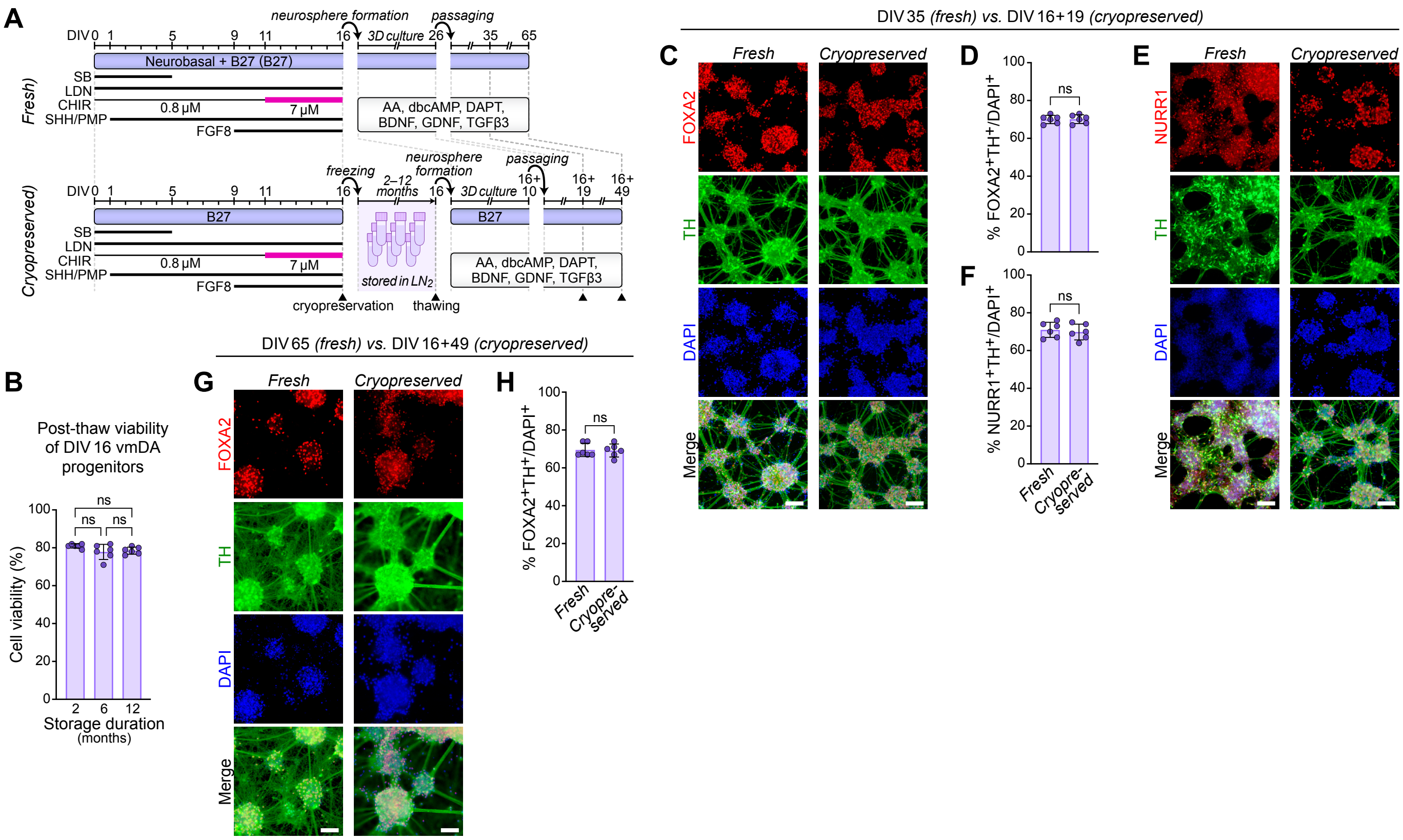

Figure S7. Cryopreserved vmDA progenitors retained viability and neurogenic capacity after long-term storage. (**A**) Schematic illustrating parallel differentiation workflows for fresh and cryopreserved vmDA lineage cells. vmDA progenitors were cryopreserved at DIV 16, stored in liquid nitrogen for 2–12 months, thawed, and subsequently subjected to the same neurosphere-based neuronal differentiation protocol used for fresh progenitors. (**B**) Post-thaw viability of vmDA progenitors assessed after the indicated storage durations (n = 6 biological replicates). (**C,D**) Representative immunofluorescence images of neurons at DIV 35 stained for FOXA2 and TH, with quantification of FOXA2/TH double-positive neurons among total DAPI$^+$ cells (n = 6 biological replicates). (**E,F**) Representative immunofluorescence images of neurons at DIV 35 stained for NURR1 and TH, with quantification of NURR1/TH double-positive neurons (n = 6 biological replicates). (**G,H**) Representative immunofluorescence images of neurons at DIV 65 stained for FOXA2 and TH, with quantification of FOXA2/TH double-positive neurons (n = 6 biological replicates). Arrowheads indicate the time points for downstream experiments. Scale bars, 100 µm. Data are presented as mean ± SD. Statistical significance was assessed by one-way ANOVA with Tukey's multiple-comparison test (**B**) or two-tailed unpaired Student's t-test (**D,F,H**). ns, not significant.

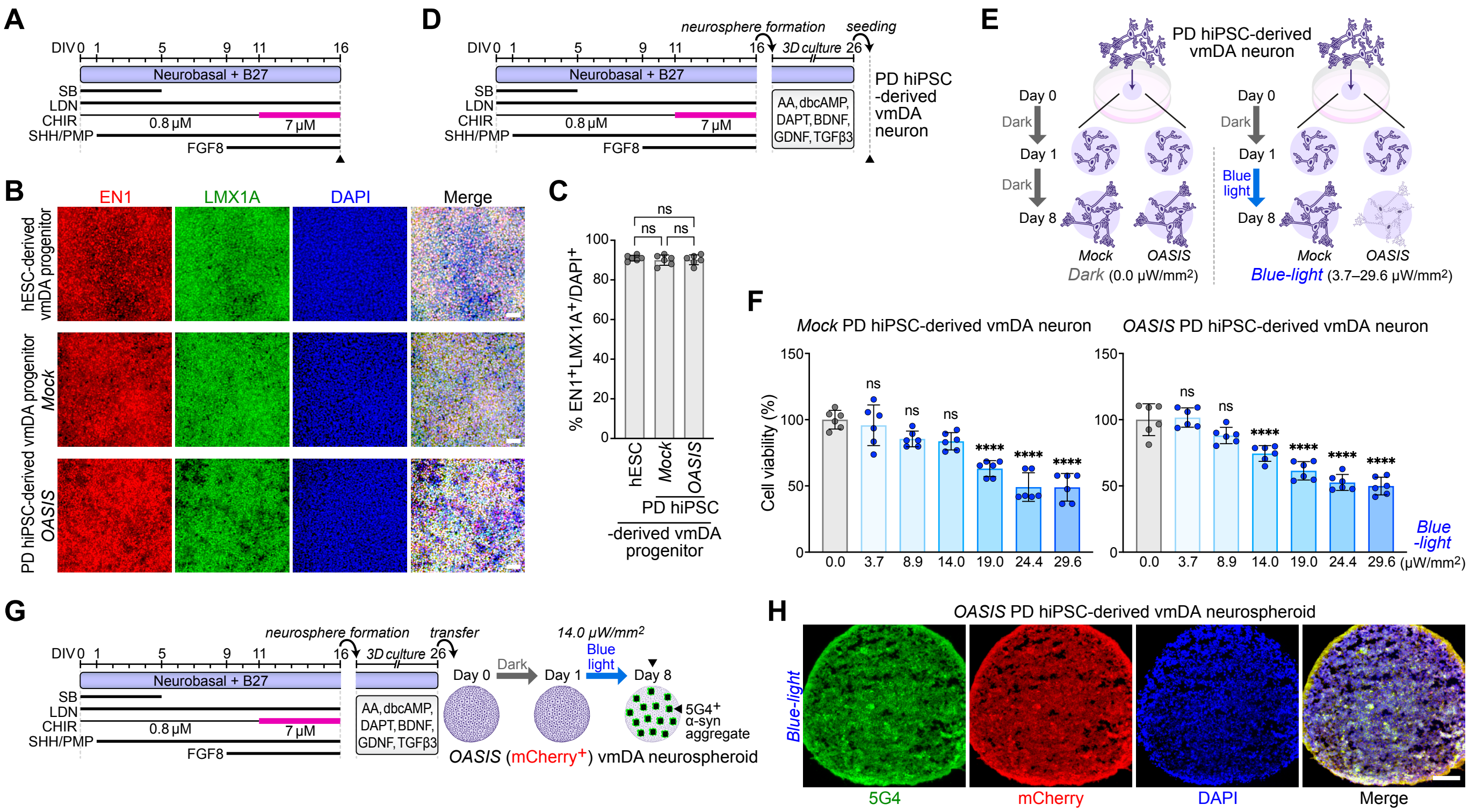

Figure S8. ***OASIS*** **did not alter vmDA progenitor specification under dark conditions but conferred light-dependent proteopathy in PD hiPSC-derived vmDA neurons.** (**A**) Schematic of vmDA progenitor differentiation from hPSCs. (**B,C**) Representative immunofluorescence images of progenitors at DIV 16 stained for EN1 and LMX1A, with quantification of EN1/LMX1A double-positive progenitors among total DAPI$^+$ cells (n = 6 biological replicates). (**D**) Schematic of vmDA neuronal differentiation and neurosphere-based maturation. (**E**) Experimental timeline for *Mock* and *OASIS* PD hiPSC-derived vmDA neurons cultured under *Dark* or *Blue-light* (3.7–29.6 μW/mm^2 at 470 nm, 0.17 Hz, 0.5 s) conditions. (**F**) Cell viability of *Mock* and *OASIS* vmDA neurons following exposure to increasing blue-light intensities (n = 6 biological replicates). Statistical comparisons were performed between the *Dark* condition (0 μW/mm^2 at 470 nm) and each blue-light intensity. (**G**) Schematic illustrating the generation of *OASIS* PD hiPSC-derived vmDA neurospheroids and the experimental paradigm for blue-light stimulation. vmDA progenitors were differentiated to DIV 16, subjected to neurosphere formation and three-dimensional culture, transferred at DIV 26, maintained under dark conditions for 24 h, and subsequently cultured for 7 days under blue-light illumination (14 μW/mm^2 at 470 nm, 0.17 Hz, 0.5 s) to induce pathological α-synuclein (α-syn) aggregation. (**H**) Representative images of cryosectioned *OASIS* vmDA neurospheroids subjected to the Blue-light condition, stained for 5G4 (pathological α-syn aggregates) and mCherry (opto-α-syn). Arrowheads indicate the time points for downstream experiments. Scale bars, 100 μm. Data are presented as mean ± SD. Statistical significance was determined by one-way ANOVA with Tukey's multiple-comparison test. ns, not significant; ****$p < 0.0001$.

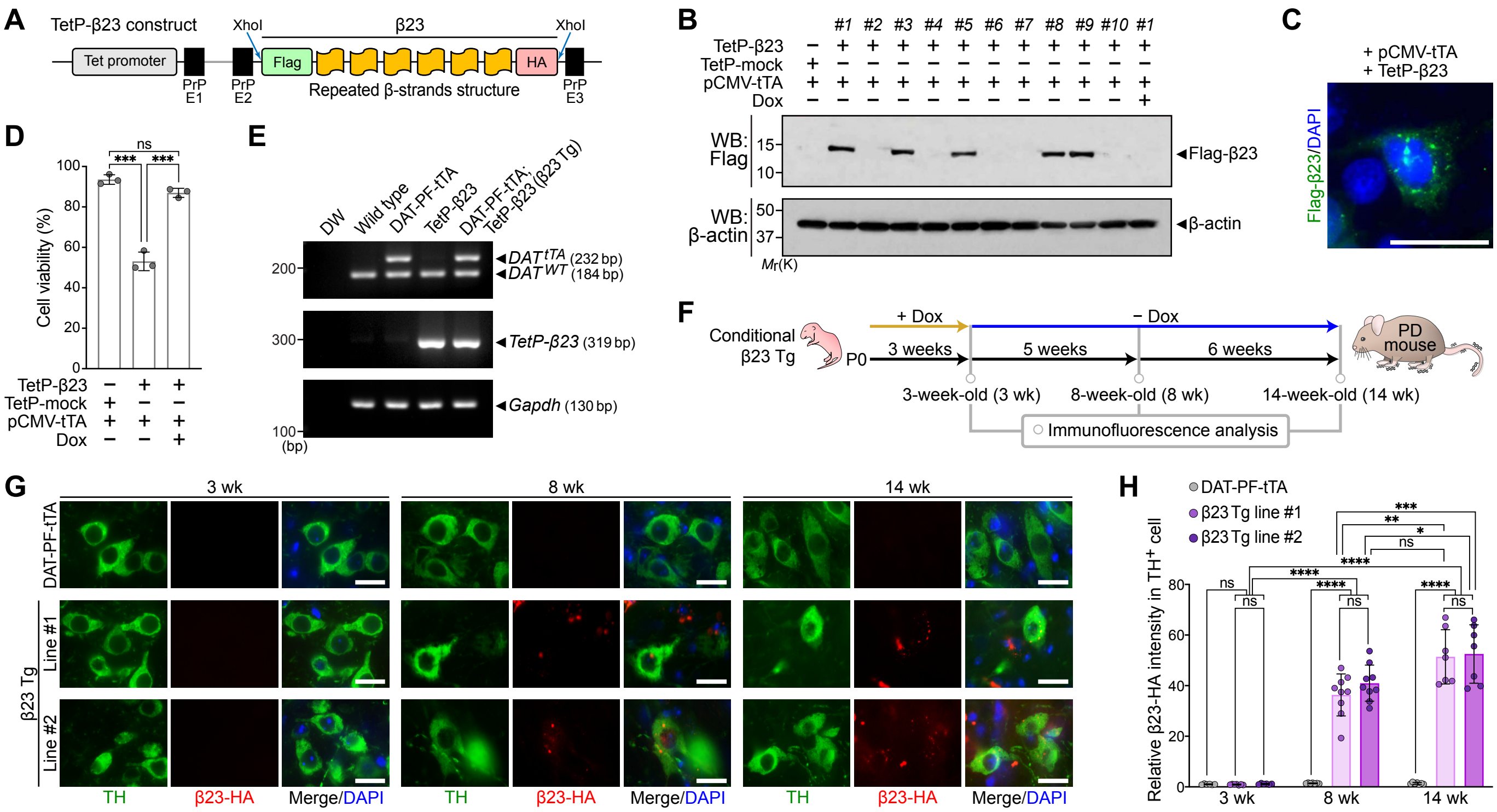

Figure S9. Generation and characterization of Tet-off conditional transgenic mice expressing the amyloid-mimic β23 in dopaminergic neurons. (**A**) Schematic of the TetP-β23 transgenic construct. The TetP vector contains prion protein (PrP) exons (E1–E3) and introns, and the β23 transgene—composed of six repeated β-strand motifs with an N-terminal Flag tag and a C-terminal HA tag—was inserted into the XhoI site of the TetP construct. (**B**) Representative Western blot analysis of Flag-tagged β23 expression in HEK293T cells co-transfected with pCMV-tTA and either TetP-mock or individual TetP-β23 constructs #1–#10. The final lane shows suppression of β23 expression following 100 ng/mL doxycycline (Dox) treatment in cells co-transfected with pCMV-tTA and TetP-β23 construct #1 (hereafter TetP-β23). (**C**) Representative immunofluorescence image showing Flag-β23^{+} puncta (green) in HEK293T cells co-transfected with pCMV-tTA and TetP-β23. Nuclei were counterstained with DAPI (blue). (**D**) Cell viability of HEK293T cells co-transfected with pCMV-tTA and TetP-β23, assessed by trypan blue exclusion (n = 3 biological replicates). (**E**) Representative genotyping PCR confirming the presence of DAT-PF-tTA and TetP-β23 transgenes in the indicated mouse groups. Genomic DNA extracted from tail biopsies was used as the PCR template, with *Gapdh* amplification serving as an internal control. Littermate DAT-PF-tTA mice were used as controls, and double-positive DAT-PF-tTA;TetP-β23 mice were designated as β23 Tg. (**F**) Experimental timeline for Tet-off–regulated β23 induction and downstream histological analyses. β23 expression was suppressed by a Dox-containing diet (200 mg Dox per kg diet) during development and induced at 3 weeks of age by switching to regular chow, followed by tissue collection and histological analyses at the indicated time points. (**G**) Representative immunofluorescence images showing TH (green) and β23-HA (red) in SNpc sections from littermate DAT-PF-tTA control mice and β23 Tg mice [lines #1 and #2] at 3, 8, and 14 weeks of age. Nuclei were counterstained with DAPI (blue). (**H**) Quantification of relative β23-HA signal intensity within dopaminergic neurons from littermate DAT-PF-tTA control mice and β23 Tg mice at the indicated ages (3 weeks, n = 6 mice for DAT-PF-tTA, n = 7 mice for β23 Tg line #1, n = 6 mice for β23 Tg line #2; 8 weeks, n = 7 mice for DAT-PF-tTA, n = 9 mice for β23 Tg [lines #1 and #2]; 14 weeks, n = 7 mice for DAT-PF-tTA, n = 7 mice for β23 Tg [lines #1 and #2]). Scale bars, 20 μm. Data are presented as mean ± SD. Statistical significance was determined by two-way ANOVA with Tukey's multiple-comparison test. ns, not significant; *$p < 0.05$, **$p < 0.01$, ***$p < 0.001$, ****$p < 0.0001$.

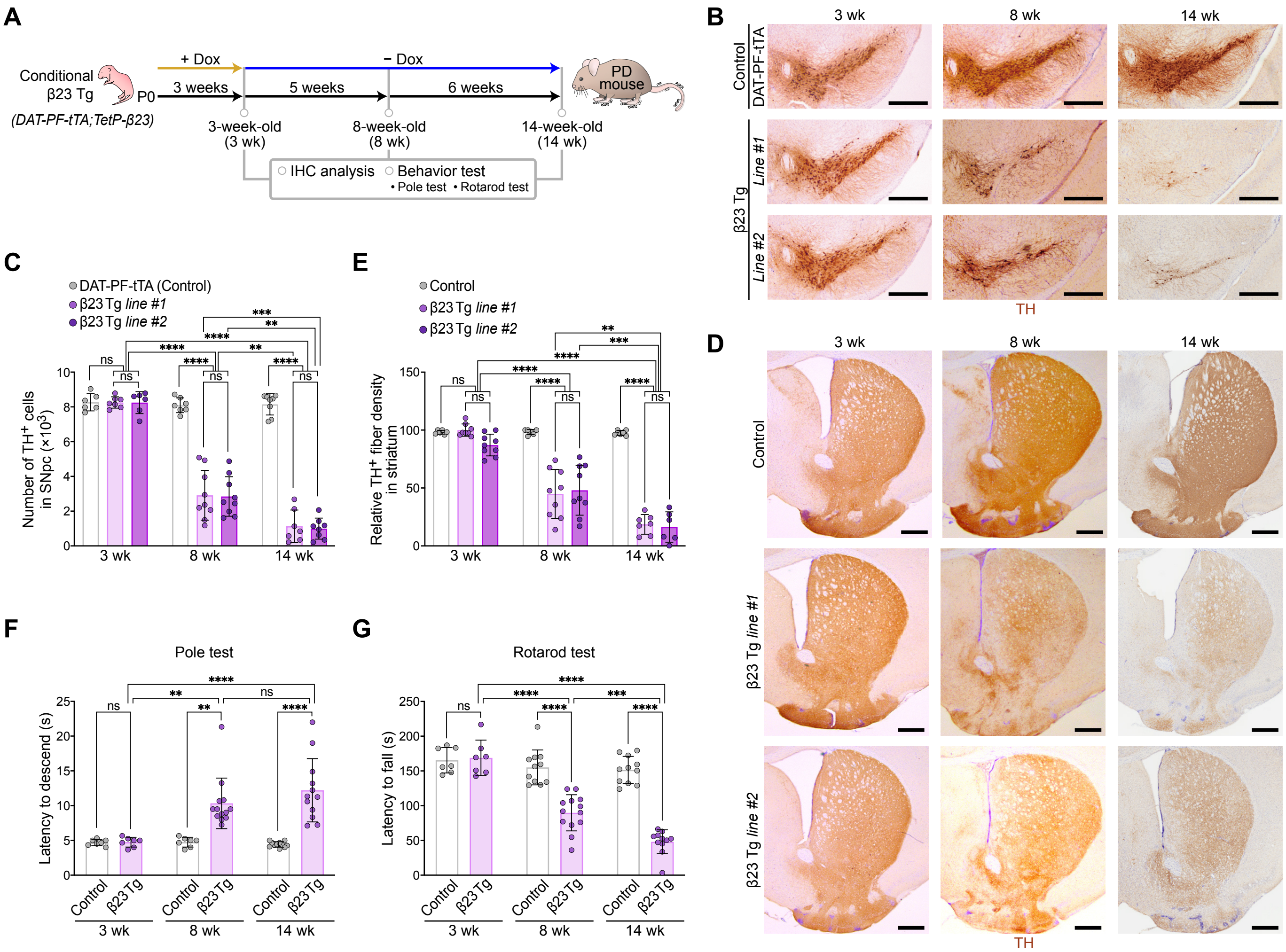

Figure S10. Dopaminergic β23 expression drove progressive nigrostriatal degeneration and motor impairment. (**A**) Schematic illustrating the experimental timeline for conditional β23 expression in DAT-PF-tTA;TetP-β23 (β23 Tg) mice. β23 expression was suppressed by feeding doxycycline (Dox)-containing diet (200 mg Dox per kg diet) during development and induced by Dox withdrawal at 3 weeks of age, followed by immunohistochemical analyses and behavioral testing at 3, 8, and 14 weeks of age. (**B**) Representative TH immunohistochemistry images of coronal ventral midbrain sections from littermate DAT-PF-tTA control mice and β23 Tg mice [lines #1 and #2] at 3, 8, and 14 weeks of age. Sections were counterstained with Nissl. (**C**) Unbiased stereological quantification of TH^+ neurons in the SNpc (3 weeks, n = 6 mice for control, n = 7 mice for β23 Tg line #1, n = 6 mice for β23 Tg line #2; 8 weeks, n = 7 mice for control, n = 9 mice for β23 Tg [lines #1 and #2]; 14 weeks, n = 8 mice for control, n = 7 mice for β23 Tg line #1, n = 8 mice for β23 Tg line #2). (**D**) Representative TH immunohistochemistry images showing dopaminergic fiber innervation in the striatum from littermate control mice and β23 Tg mice [lines #1 and #2] at the indicated ages. Sections were counterstained with Nissl. (**E**) Quantification of relative TH^+ fiber density in the striatum (3 weeks, n = 6 mice for control, n = 8 mice for β23 Tg line #1, n = 9 mice for β23 Tg line #2; 8 weeks, n = 6 mice for control, n = 9 mice for β23 Tg line #1, n = 7 mice for β23 Tg line #2; 14 weeks, n = 6 mice for control, n = 9 mice for β23 Tg line #1, n = 6 mice for β23 Tg line #2). (**F**) Assessment of bradykinesia by pole test in littermate control mice and β23 Tg mice at the indicated ages (3 weeks, n = 7 mice for control, n = 7 mice for β23 Tg; 8 weeks, n = 7 mice for control, n = 13 mice for β23 Tg; 14 weeks, n = 11 mice for control, n = 12 mice for β23 Tg). (**G**) Accelerating rotarod test assessing motor coordination in littermate control mice and β23 Tg mice at the indicated ages (3 weeks, n = 7 mice for control, n = 7 mice for β23 Tg; 8 weeks, n = 11 mice for control, n = 13 mice for β23 Tg; 14 weeks, n = 11 mice for control, n = 11 mice for β23 Tg). Scale bars, 500 μm. Data are presented as mean ± SD. Statistical significance was determined by two-way ANOVA with Tukey's multiple-comparison test. ns, not significant; $^{**}p < 0.01$, $^{***}p < 0.001$, $^{****}p < 0.0001$.

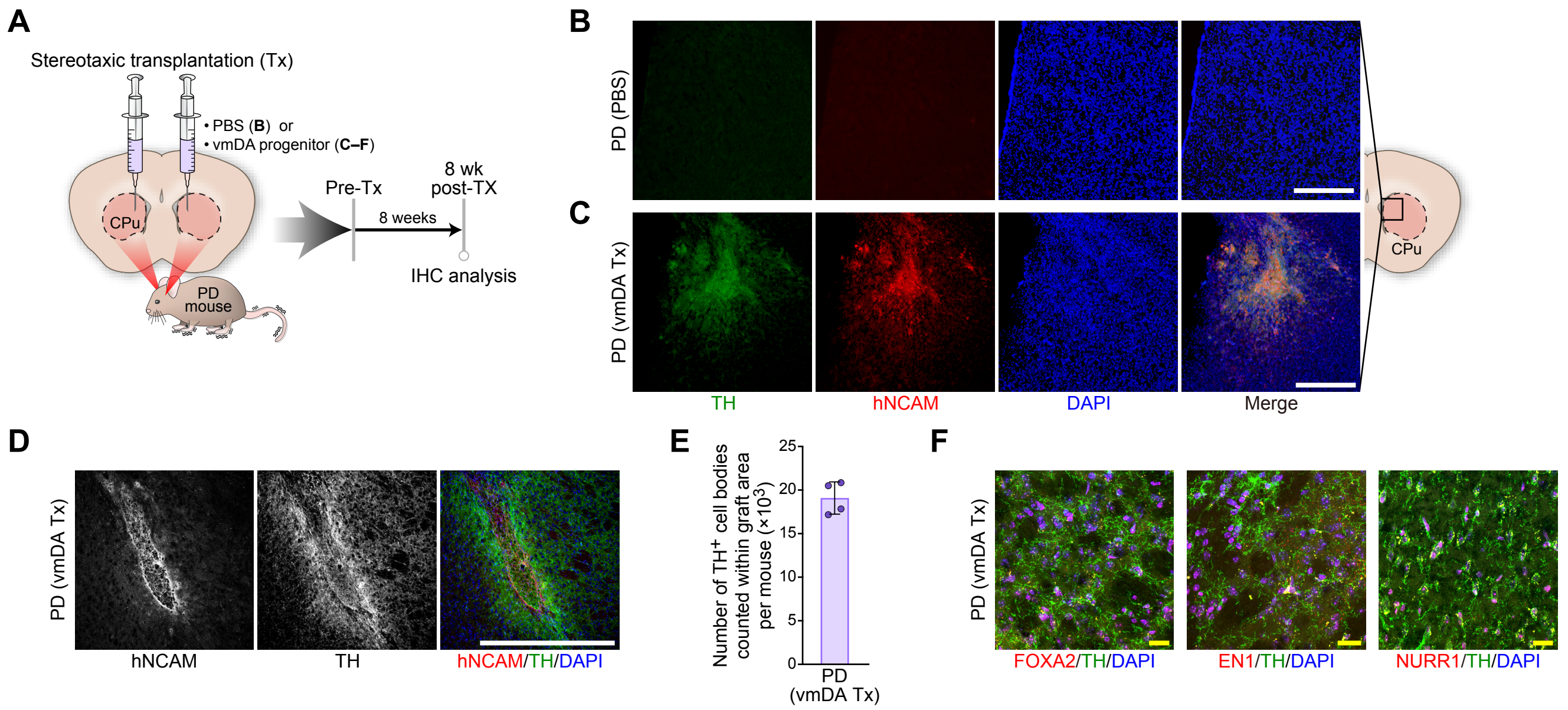

Figure S11. Verification of survival and dopaminergic differentiation of transplanted vmDA progenitors in a proteopathy-based PD mouse model. (A) Schematic illustrating the stereotaxic transplantation (Tx) paradigm. hESC-derived vmDA progenitors differentiated to DIV 16 using the optimized protocol were bilaterally transplanted into the striatum of 14-week-old DAT-PF-tTA;TetP-β23 mice, a proteopathy-based PD mouse model, followed by immunohistochemical analyses at 8 weeks post-Tx. (**B**) Representative immunofluorescence images of striatal sections from PBS-injected PD mice stained for TH (green), hNCAM (red), and DAPI (blue). (**C**) Representative immunofluorescence images of striatal sections from vmDA progenitor-transplanted PD mice stained for TH (green), hNCAM (red), and DAPI (blue), showing survival of graft-derived dopaminergic neurons. (**D**) Separate hNCAM and TH channels corresponding to the merged image shown in Figure 6C (upper left); the merged image is also shown here for reference. (**E**) Quantification of TH$^+$ cell bodies counted within the graft area for each mouse (*n* = 4 mice). Data are presented as mean ± SD. (**F**) Representative high-magnification immunofluorescence images of graft-containing sections stained for FOXA2/TH, EN1/TH, and NURR1/TH, showing nuclear localization of the indicated transcription factors in TH$^+$ cells within the graft region. Nuclei were counterstained with DAPI. Scale bars, 500 μm (white) and 20 μm (yellow).

Supplemental Tables

Supplemental Table Legends

Table S1. ORA results for Gene Ontology (GO) terms (biological process, cellular component, and molecular function) across Group A, Group B, and Group C.

Table S2. ORA results for KEGG pathways across Group A, Group B, and Group C.

Table S3. ORA results for MSigDB gene sets across Group A, Group B, and Group C.

Table S4. ORA results for Reactome pathways across Group A, Group B, and Group C.

Table S5. RNA-seq expression matrices (raw counts and FPKM) across DIV 0, 9, 16, and 35, with genes indexed by official gene symbols.

Table S6. Cluster-specific gene lists used for over-representation analysis (ORA), including official gene symbols for each cluster.
